# Fluoxetine pharmacokinetics and tissue distribution suggest a possible role in reducing SARS-CoV-2 titers

**DOI:** 10.12688/f1000research.53275.1

**Published:** 2021-06-16

**Authors:** Andy R. Eugene

**Affiliations:** 1 Independent Neurophysiology Laboratory, Department of Psychiatry, Medical University of Lublin, Lublin Voivodship, 20-059, Poland; 2 Institute for the Study of Child Development, Department of Pediatrics, Robert Wood Johnson Medical School, Rutgers University, New Brunswick, New Jersey, 08901, USA

**Keywords:** Prozac, Sarafem, SARS- COV- 2, COVID-19, antidepressant, pharmacokinetics, dose, lungs

## Abstract

**Background. ** Recent in vitro studies have shown fluoxetine inhibits the severe acute respiratory syndrome coronavirus 2 (SARS-CoV-2) pathogen, including variants B.1.1.7 and B.1.351, SARS-CoV-2 spike mutations (E484K, K417N, N501Y), and one retrospective clinical study reported fluoxetine exposure at a median dose of 20 mg in patients with the SARS-CoV-2 coronavirus disease 2019 (COVID-19) had a significantly lower risk of intubation and death. The aim of this study is to conduct in silico population pharmacokinetic dosing simulations to quantify the percentage of patients achieving a trough level for the effective concentration resulting in 90% inhibition (EC90) of SARS-CoV-2 as reported in Calu-3 human lung cells.

**Methods.**  Population pharmacokinetic parameter estimates for a structural one-compartment model with first-order absorption were used to simulate fluoxetine pharmacokinetic data. A population of 1,000 individuals were simulated at standard fluoxetine doses (20 mg/day, 40 mg/day, and 60 mg/day) to estimate the percentage of the patients achieving a trough plasma level for the EC90 SARS-CoV-2 inhibitory concentration for a 10 day treatment period. All analyses were conducted via statistical programming in R.

**Results.**  Standard fluoxetine antidepressant doses resulted in a range of 81% to 97% of the patient population achieving a trough target plasma concentration of 23.2 ng/ml at day 10 and translates to a lung-tissue distribution coefficient of 60-times higher (EC90 of 4.02 mM). At a dose of 40 mg per day, at least 87% of patients will reach the trough target EC90 concentration within three days.

**Conclusion.** Overall, the findings of this population pharmacokinetic dosing study corroborates in vitro and observational clinical studies reporting the first selective serotonin reuptake inhibitor fluoxetine inhibits the SARS-CoV-2 pathogen at commonly treated doses in the practice of psychiatry.

## Introduction

The selective serotonin reuptake inhibitor (SSRI) fluoxetine is a racemic mixture of two stereoisomers, *R*-fluoxetine and *S*-fluoxetine, and maintains regulatory approvals for a wide-array of clinical indications in the practice of psychiatry. Two recent *in vitro* studies showed fluoxetine inhibits replication of the Severe Acute Respiratory Coronavirus-2 (SARS-CoV-2) pathogen ( Schloer *et al*., 2020; Zimniak *et al*., 2020, 2021). Specifically, Zimniak *et al*. reported that following a three-day incubation period of fluoxetine in Vero cells, inoculated at a multiplicity of infection (MOI) of 0.5, resulted in the median maximal effective concentration (EC50) of 387 ng/ml (1.1 μM) and further found a concentration of 800 ng/ml (2.3 μM) significantly inhibited SARS-CoV-2 replication ( Zimniak *et al*., 2020, 2021). Similarly, Schloer *et al*. found that fluoxetine significantly decreases SARS-Cov-2 titers, after a 48-hour incubation period, in both African green monkey kidney epithelial Vero E6 cells (EC50 = 0.69 μM and 90% maximal effective concentration [EC90] = 1.81 μM, MOI = 0.01) and human-lung Calu-3 cells (EC50 = 0.82 μM and EC90 = 4.02 μM, MOI = 0.1) ( Schloer *et al*., 2020). Taken together these *in vitro* studies prove in a dose-dependent manner that the SSRI fluoxetine inhibits the SARS-CoV-2 pathogen known to cause the worldwide pandemic, the novel coronavirus disease 2019 (COVID-19).

Considering the COVID-19 clinical symptoms affecting the lungs, fluoxetine lung concentrations would be an important factor to consider when interpreting any study results. Johnson *et al*. reported human-tissue concentrations of fluoxetine in airline pilots in whole-blood ranged from 0.021–1.4 μg/ml and lung concentrations ranged from 1.56 μg/ml to 51.9 μg/ml, leading to a fluoxetine distribution coefficient of 60 ( Johnson, Lewis & Angier, 2007). Clinically, the fluoxetine SARS-CoV-2 *in vitro* findings were corroborated by Hoertel *et al*. who showed in a multicenter observational retrospective cohort study of patients who were treated with fluoxetine and diagnosed with COVID-19, experienced a lower risk of intubation and death (hazard ratio = 0.32; 95% confidence interval, 0.14–0.73, p = 0.007) at a median fluoxetine dose of 20 mg (standard deviation [SD] = 4.82) ( Hoertel *et al*., 2020). In this context, the aim of this study is to conduct *in silico* population pharmacokinetic dosing simulations to quantify the percentage of patients expected to achieve the *trough* effective concentration resulting in 90% inhibition of SARS-CoV-2.

## Methods

### Pharmacokinetic model

Pharmacometric model estimates for differential equation parameters and respective variances for a structural one-compartment pharmacokinetic model with first-order absorption were used to simulate fluoxetine concentration-time data. Model estimates were derived from drug plasma concentrations in 25 females taking a mean dose of 29.4 mg (7.5–80 mg/day) when fluoxetine plasma levels were at steady-state due to being collected for analysis at a minimum median time of fluoxetine treatment of greater than 40 days ( Tanoshima *et al*., 2014). The following parameters were used: volume of distribution (Vd) value of 20.5 liters (variance [ω], 1.24), clearance rate (CL) value of 13.3 liters/hour (ω = 0.052), and absorption rate (Ka) of 0.016 (1/hour) (ω = 0.231) ( Tanoshima *et al*., 2014).

### Target fluoxetine plasma concentration to achieve EC90 lung concentration

The molecular weight of fluoxetine hydrochloride is 345.8 g/mol and the reported EC50 (0.82 μM) and EC90 (4.02 μM) values from the Schloer *et al*. study are equivalent to EC50 = 283.6 ng/ml and EC90 = 1390.1 ng/ml, respectively. The fraction of fluoxetine bound in human plasma is 94%, which leaves only 6% of the compound being unbound in human plasma ( Sommi, Crismon & Bowden, 1987). Despite fluoxetine being highly protein bound, a study by Mantinieks *et al.* reported in paired fluoxetine concentrations of antemortem and postmortem cases (n = 18), fluoxetine has a human whole-blood to plasma ratio of 0.8-1.0, meaning that the whole-blood concentration is actually less than plasma or has up to a 1:1 ratio ( Mantinieks *et al*., 2020). Further, Mantinieks *et al.* found the postmortem (range: 0.031–1.4 mg/L) to antemortem (range: 0.018–0.51 mg/L) fluoxetine drug concentration ratio as 1.8, but was not statistically significant as the p-value >0.05 and thus the 1.8 ratio is not applicable to this study ( Mantinieks *et al*., 2020). Therefore, this study will directly translate the simulated plasma concentrations and apply the tissue distribution coefficients from the Johnson *et al.* study and the original preprint version of the manuscript is updated to account for the findings from Mantinieks *et al.* ( Johnson, Lewis & Angier, 2007; Eugene, 2020; Mantinieks *et al*., 2020). Lastly, for all calculations, the *trough* target plasma concentration is referenced from the Schloer *et al*. study who reported after a 48-hour incubation period in Calu-3 lung cells the 90% maximal effective concentration is 4.02 μM ( Schloer *et al*., 2020), which is significantly higher than the EC90 in Vero E6 cells (1.81 μM) and EC50 results from Zimniak *et al*. and the Schloer *et al*. studies ( Schloer *et al*., 2020; Zimniak *et al*., 2020, 2021).

### Dosing simulations

To estimate the percentage of patients from a population of one thousand simulated patients who would achieve the *trough* target EC90 concentration, pharmacokinetic dosing of fluoxetine consisted of three dosing trials of fluoxetine: 20 mg/day, 40 mg/day, and lastly 60 mg/day.

### Software and statistics

All pharmacokinetic dosing simulations are conducted with a population of 1,000 patients using *mrgsolve* and pharmacokinetic parameter estimates using *PKNCA* in R version 3.6.3 ( R Core Team, 2015). The overall R script has been adapted from a study published in Clinical Pharmacology and Therapeutics using hydroxychloroquine ( Al-Kofahi *et al*., 2020; Eugene, 2021). Statistical results providing percentage estimates are calculated from *trough* concentrations of patients achieving the effective concentrations and is referenced from the Schloer *et al*. study reporting the EC90 value in human-lung Calu-3 cells ( Schloer *et al*., 2020).

## Results

**Figure 1.**
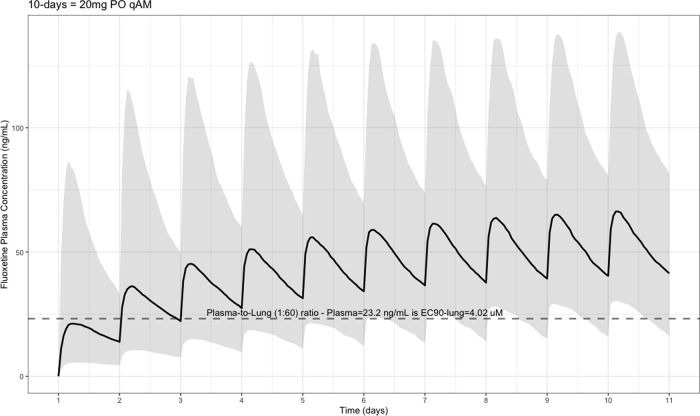
Fluoxetine population (n = 1,000) dosing simulation results for an oral dose of *20 mg/day* for 10 days. The shaded regions illustrate the 10th (lower) and 90th (upper) percentiles with the solid line within the shaded region representing the median fluoxetine concentration. The dashed horizontal line depicts the effective concentration resulting in 90% inhibition (EC90) of SARS-Cov-2 that will result in 60-times higher level in the lungs.

**Figure 2.**
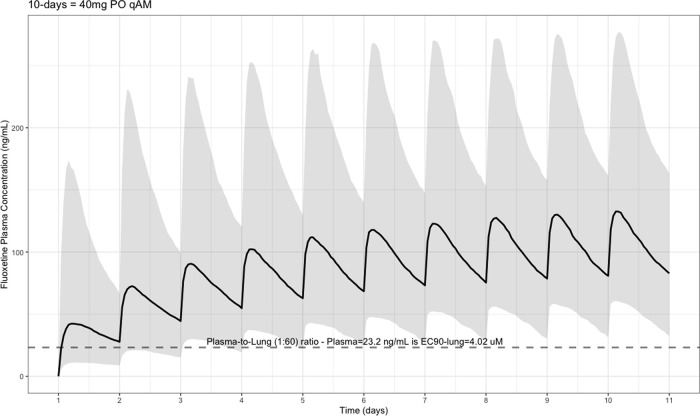
Fluoxetine population (n = 1,000) dosing simulation results for an oral dose of *40 mg/day* for 10 days. The shaded regions illustrate the 10th (lower) and 90th (upper) percentiles with the solid line within the shaded region representing the median fluoxetine concentration. The dashed horizontal line depicts the effective concentration resulting in 90% inhibition (EC90) of SARS-Cov-2 that will result in 60-times higher level in the lungs.

**Figure 3.**
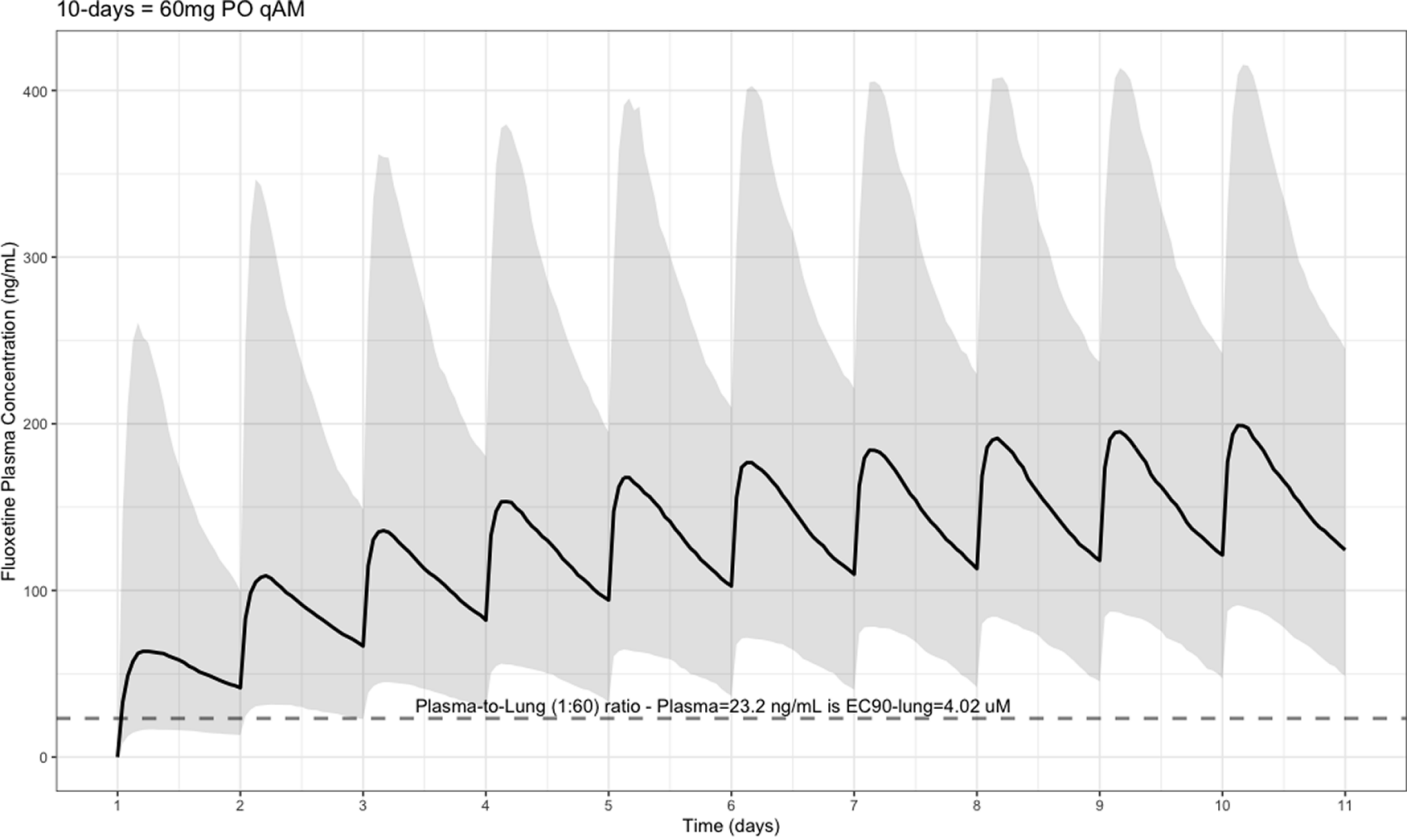
Fluoxetine population (n = 1,000) dosing simulation results for an oral dose of *60 mg/day* for 10 days. The shaded regions illustrate the 10th (lower) and 90th (upper) percentiles with the solid line within the shaded region representing the median fluoxetine concentration. The dashed horizontal line depicts the effective concentration resulting in 90% inhibition (EC90) of SARS-Cov-2 that will result in 60-times higher level in the lungs.

The EC90 target fluoxetine lung concentration is 1390.1 ng/ml [4.02 μM] and 1/60 of this concentration is the new EC90-plasma concentration of 23.2 ng/ml [0.067 μM]. The percentage of the 1,000 simulated patients are illustrated in Figure 1 (20 mg/day), Figure 2 (40 mg/day), and Figure 3 (60 mg/day) with a horizontal dashed-line throughout the pharmacokinetic dosing figures showing the required *trough* EC90-plasma level of 23.2 ng/ml that translates to the EC90 level of 1390.1 ng/ml [4.02 μM] in the lungs.

Figure 1 shows the concentration-time data for a fluoxetine dose of 20 mg per day and results in the maximum plasma concentration (Cmax) with a geometric mean (geometric coefficient of variation, CV%) of 65.8 ng/mL (CV=70.2%), median time at maximum concentration (Tmax) of 220 hours (range, 49–220), area under the concentration-time curve (AUC _0➔Last_) of geometric mean from baseline to 10 days of 10,200 ng•hour/ml, and a half-life (t ½) – expressed as arithmetic mean (standard deviation, SD) – of 84.7 hours (SD = 181). These aforementioned pharmacokinetic results translate to 24% of the population reaching the target concentration at the end of day one and 81% of the population achieving the target *trough* EC90 concentration by end of day 10. Figure 2 shows at a dose of 40 mg per day, the Cmax is 132 ng/mL (CV = 68%), Tmax of 220 hours (range, 49–220), AUC _0➔Last_ is 20,500 ng•hour/ml, and population t ½ is 81.4 (SD = 113), which is interpreted as 59% of the population achieving the EC90 *trough* target at day one and 93% by day 10. Moreover, Figure 3 shows a patient population treated with fluoxetine at 60 mg daily results in a Cmax of 191 ng/mL (CV = 71%), 220 hours (range, 49–232), AUC _0➔Last_ 29,700 ng•hour/ml, and t ½ of 85.4 (SD = 209) allowing 74% of the population to reach the target *trough* concentration threshold on day one and 97% by day 10 of fluoxetine treatment. Table 1 provides an overview of the pharmacokinetics and pharmacodynamics with blood levels (ng/ml and μM) in plasma as well as calculated organ concentrations (whole-blood, lung, brain, heart, liver, spleen, and kidney) as well as the percent of the population achieving *trough* EC90 target during a treatment period of 10 days. All underlying fluoxetine pharmacokinetic study data in an.xlsx format, the one-compartment population pharmacokinetic model file in C++ format, and the R programming script are available ( Eugene, 2021).

**Table 1.** Fluoxetine pharmacokinetics and pharmacodynamics showing blood levels (ng/mL and μM) and percent of population achieving plasma *trough* concentration of 23.2 ng/ml leading to a lung-EC90 target of 4.02 μM during a treatment period of 10-days in humans.

	Fluoxetine tissue distribution concentrations at standard antidepressant doses		
	20 mg/day	40 mg/day	60 mg/day
	Cmax ng/ml [μM]	Cmax ng/ml [μM]	Cmax ng/ml [μM]
Plasma	65.5 [0.19] (CV = 70%)	132 [0.38] (CV = 68%)	191 [0.55] (CV = 71%)
Whole-Blood	65.5 [0.19]	132 [0.38]	191 [0.55]
Lung	3930 [11.4]	7920 [22.9]	11460 [33.1]
Brain	982.5 [2.8]	1980 [5.7]	2865 [8.3]
Heart	655 [1.9]	1320 [3.8]	1910 [5.5]
Liver	2489 [7.2]	5016 [14.5]	7258 [21.0]
Spleen	1310 [3.8]	2640 [7.6]	3820 [11.0]
Kidney	589.5 [1.7]	1188 [3.4]	1719 [5.0]

	Percent of population (n = 1,000) achieving the *trough* SARS-CoV-2 EC90 target in lungs		
Time Point	20 mg/day	40 mg/day	60 mg/day
Day 1	24%	59%	74%
Day 2	47%	79%	90%
Day 3	60%	87%	93%
Day 4	67%	90%	94%
Day 5	72%	90%	95%
Day 6	76%	92%	95%
Day 7	78%	92%	96%
Day 8	80%	93%	96%
Day 9	81%	93%	96%
Day 10	81%	93%	97%

EC90: effective concentration inhibiting 90% of SARS-CoV-2 in Calu-3 lung cells reported as 4.02 uM ( Schloer *et al*., 2020). Maximum plasma concentration (Cmax) is expressed as geometric mean (geometric coefficient of variation, CV%). Fluoxetine tissue distribution coefficients (60 for lung, 15 for brain, 10 for heart, 38 for liver, 20 for spleen, and 9 for kidneys) as reported ( Johnson, Lewis & Angier, 2007).

## Discussion

According to the United States Food and Drug Administration (FDA) Adverse Events Reporting System (FAERS) during the window period of 1982 to June 30, 2020, fluoxetine was reported to have a total of 79,929 cases, 62,948 serious cases, and 10,043 end of life cases ( https://www.fda.gov/drugs/questions-and-answers-fdas-adverse-event-reporting-system-faers/fda-adverse-event-reporting-system-faers-public-dashboard). Females represented 58% of the adverse drug reactions (ADRs), males represented 27% of the ADRs, and 15% of the ADRs did not specify a gender. The most common adverse drug event reported for fluoxetine is Drug Interaction and amounts to 3,798 cases (4.75% of total). Given this information, drug interactions associated with fluoxetine are due to inhibition of the cytochrome P450 (CYP) system. Specifically, CYP2C19 and CYP2D6 may have interactions such as in patients taking tamoxifen for breast cancer by inhibiting conversion to the active endoxifen metabolite via CYP2D6 or in cases of clopidogrel in cardiology by inhibiting the conversion of clopidogrel to the active 2-oxo-clopidogrel metabolite ( Spina, Trifirò & Caraci, 2012; Eugene, 2019).

Extrapolating from *in vitro* to *in vivo* concentrations are dependent on intracellular versus extracellular concentrations, as well as the methodology of quantifying either whole blood versus plasma concentrations in human pharmacokinetic studies. The EC50 and EC90 target concentrations represent the extracellular fluoxetine concentrations in the SARS-CoV-2 cell culture media. As COVID-19 is known to affect the brain during active infection and in post-COVID-19 states, adequate brain concentrations would be clinically important in patients who may experience depression. Bolo *et al*. reported fluoxetine brain concentrations, at steady-state, using fluorine magnetic spectroscopy and showed fluoxetine concentrations were 10-times higher in the brain than in human plasma ( Bolo *et al*., 2000). Specifically, Bolo *et al*. found in study participants taking oral doses (10mg, n = 1; 20 mg, n = 1; 40 mg, n = 2) with a treatment period ranging from three months to 12-months that fluoxetine human brain concentrations were 13 μM (SD = 7) versus 1.73 μM (SD = 1.00) in human plasma fluoxetine ( Bolo *et al*., 2000). In comparison, Johnson *et al*. found the coefficients for tissue distribution of fluoxetine relative to whole blood was: 60 for lung, 15 for brain, 10 for heart, 38 for liver, 20 for spleen, and 9 for kidneys ( Johnson, Lewis & Angier, 2007).

As patients recover from the acute COVID-19 symptoms, long-term sequelae are being documented and in one of the post-SARS-Cov-2 infection studies in young patients, 92% were found to have ongoing cardiorespiratory symptoms with organ dysfunction and impairment in the lungs (33%), heart (32%), kidneys (12%) ( Dennis *et al*., 2020). In another post-COVID-19 syndrome study, 96% of the patients were female and experienced statistically significant exercise intolerance, dyspnea, and chest pain when compared to those not diagnosed with COVID-19 ( Walsh-Messinger *et al*., 2020). Moreover, Walsh-Messinger *et al*. found patients with post-COVID-19 syndrome had higher ratings of depression subscale markers of altered sleep and thinking, but depression severity was not significantly different with patients not diagnosed with COVID-19 ( Walsh-Messinger *et al*., 2020).

Direct clinical translation of this current pharmacokinetic study corroborates with a retrospective multicenter observational study by Hoertel *et al*., who found a median fluoxetine dose of 20mg/day resulted in a significantly lower risk of intubation and death in a population composed of 63% women and 37% men ( Hoertel *et al*., 2020). Comparing the Hoertel *et al*. and Zimniak *et al*. publications, Hoertel *et al*. found that in addition to fluoxetine, venlafaxine (median dose of 75mg/day) and escitalopram (median dose of 10mg) were also associated with a lower risk of intubation and death, however, Zimniak *et al*. showed that neither escitalopram nor paroxetine inhibited SARS-CoV-2 *in vitro* ( Hoertel *et al*., 2020; Zimniak *et al*., 2020, 2021). Of note, as shown in Table 1, a 40 mg or 60 mg daily fluoxetine dose results in 90% inhibition of the SARS-CoV-2 infection due to surpassing the EC90 value of 4.02 μM as found in Calu-3 cells and the EC90 value of 1.81 μM in Vero E6 cells ( Schloer *et al*., 2020).

Antiviral properties of fluoxetine are well reported in the literature. Carpinteiro *et al*. reported that fluoxetine inhibits acid sphingomyelinase preventing infection of both cultured cells and human nasal epithelial cells in SARS-CoV-2, as well as in vesicular stomatitis virus pseudoviral particles presenting the SARS-CoV-2 spike protein ( Carpinteiro *et al*., 2020). A study by Zuo *et al*. showed fluoxetine resulted in potent inhibition of the coxsackievirus by reducing both synthesis of viral RNA and protein (EC50 of 2.3 μM) exhibiting peak antiviral properties at 6.25 μM ( Zuo *et al*., 2012). Bauer *et al*. showed, in a broad-spectrum manner, fluoxetine inhibited enterovirus (picornaviridae family) replication with the *S*-fluoxetine enantiomer exhibiting a 5-fold lower EC50 than the racemic mixture of *R-* and *S*-fluoxetine ( Bauer *et al*., 2019). Further, Bauer *et al*. found the following fluoxetine EC50 values for the following pathogens: coxsackievirus B3 (racemate-EC50 = 2.02 μM, *S*-fluoxetine-EC50 = 0.42 μM), enterovirus EV-D68 (racemate-EC50 = 1.85 μM, *S*-fluoxetine-EC50 = 0.67 μM), and *S*-fluoxetine values alone for rhinovirus HRV-A2 (EC50 = 7.95 μM) and HRV-B14 (EC50 = 6.34 μM) ( Bauer *et al*., 2019). Notably, Zimniak *et al*. found that individual stereoisomers, *R*-fluoxetine and *S*-fluoxetine, inhibited the SARS-CoV-2 viral load; however, in contrast, fluoxetine could not inhibit gene expression of the herpes simplex-1 virus, human herpes virus-8, rabies virus, nor the respiratory syncytial virus ( Zimniak *et al*., 2020, 2021). Lastly, as shown in Table 1, standard fluoxetine doses are capable of achieving the aforementioned EC50s for all of the aforementioned microbes.

A limitation of this study is associated with the previously validated fluoxetine pharmacometric model being in women and did not include men ( Tanoshima *et al*., 2014). However, as shown from the aforementioned FAERS data, women represented 58% of all ADR cases overall from 1982 to 2020. A significant study strength is that a study from the University of Helsinki reported fluoxetine inhibits SARS-CoV-2 variants (B.1.1.7 and B.1.351) and the spike mutations (E484K, K417N, N501Y) ( Fred *et al*., 2021). Overall, from a drug-safety perspective, prior to administering fluoxetine, a careful review of all patient medications and clinical status by a clinical pharmacologist physician would be recommended to avoid drug interactions due to fluoxetine’s ability to strongly inhibit CYP2C19 and CYP2D6 ( Hefner, 2018). Compounds that are sensitive and moderate CYP2C19 substrates (e.g. omeprazole, diazepam, lansoprazole, rabeprazole, voriconazole) and CYP2D6 substrates (e.g. dextromethorphan, eliglustat, nebivolol, tolterodine, encainide, metoprolol, propranolol, tramadol) will have an increased total area under the concentration-time curve of ≥ 5-fold drug exposure when treated with fluoxetine ( https://www.fda.gov/drugs/drug-interactions-labeling/drug-development-and-drug-interactions-table-substrates-inhibitors-and-inducers). Lastly, patients who have a pharmacogenomic profile of being a CYP2D6 Poor Metabolizer or CYP2D6 Intermediate Metabolizer should be closely monitored for potential fluoxetine side-effects; but they may also have a higher rate of achieving the target *trough* EC90 concentration at a 20 mg daily fluoxetine dose relative to CYP2D6 Normal (Extensive) Metabolizers.

## Conclusions

This study investigated fluoxetine pharmacokinetics and human organ tissue distribution which confirmed that previously published median effective concentrations and specifically the EC90 fluoxetine value inhibiting SARS-CoV-2 in Calu-3 human lung cells are achievable using standard fluoxetine doses (20mg/day, 40mg/day, and 60mg/day) and also corroborates findings from a retrospective clinical study showing fluoxetine exposure was associated with reduced risk of intubation and death. Overall, assuming patients are not treated with medications that result in drug-drug interactions with fluoxetine, a dose of 40 mg per day of fluoxetine will likely be most effective with inhibiting the SARS-CoV-2 viral titers with 59% of the population achieving the *trough* EC90 target on day one, 92% by day seven, and 93% of patient population achieving the *trough* target EC90 concentration to inhibit the SARS-CoV-2 within 10 days.

## Data availability

### Underlying data

Open Science Framework: Underlying data for ‘Fluoxetine pharmacokinetics and tissue distribution suggest a possible role in reducing SARS-CoV-2 titers’, https://doi.org/10.17605/OSF.IO/R7ND6 ( Eugene, 2021).

This project contains the following underlying data:

• Data File 1: fluoxetine_20mg_PO_QAM.xlsx
• Data File 2: fluoxetine_40mg_PO_QAM.xlsx
• Data File 3: fluoxetine_60mg_PO_QAM.xlsx

### Software availability

Open Science Framework: Software for ‘Fluoxetine pharmacokinetics and tissue distribution suggest a possible role in reducing SARS-CoV-2 titers’, https://doi.org/10.17605/OSF.IO/R7ND6 ( Eugene, 2021).

This project contains the following software:

• poppk_fluoxetine_sars_cov2_inhibition.cpp
• Fluoxetine_pharmacokinetic_sars_cov2_simulation_script.R

Data are available under the terms of the Creative Commons Zero “No rights reserved” data waiver ( CC0 1.0 Public domain dedication).
