# Fluoxetine pharmacokinetics and tissue distribution quantitatively supports a therapeutic role in COVID-19 at a minimum dose of 20 mg per day

**DOI:** 10.12688/f1000research.53275.2

**Published:** 2022-02-02

**Authors:** Andy R. Eugene

**Affiliations:** 1 Independent Neurophysiology Laboratory, Department of Psychiatry, Medical University of Lublin, Lublin Voivodship, 20-059, Poland; 2 Institute for the Study of Child Development, Department of Pediatrics, Robert Wood Johnson Medical School, Rutgers University, New Brunswick, New Jersey, 08901, USA

**Keywords:** SARS-CoV-2, COVID-19, SSRI, psychopharmacology, coronavirus, fluoxetine vs fluvoxamine, titers, repurposing

## Abstract

**Background. ** Various *in vitro* studies have shown fluoxetine inhibits multiple variants of the severe acute respiratory syndrome coronavirus 2 (SARS-CoV-2) pathogen causing the coronavirus disease 2019 (COVID-19) worldwide pandemic and multiple observational clinical studies have shown that patients receiving fluoxetine experienced clinical benefit by lowering the risk of intubation and death. The aim of this study is to conduct population pharmacokinetic dosing simulations to quantify the percentage of patients achieving a *trough* level for the effective concentration resulting in 50% (EC50) and 90% (EC90) inhibition of SARS-CoV-2 as reported in Calu-3 human lung cells.

**Methods. ** Pharmacometric parameter estimates used in this study were obtained from the U.S. FDA website from a new drug application for fluoxetine hydrochloride. A population of 1,000 individuals were simulated at standard fluoxetine antidepressant doses (20 mg/day, 30 mg/day, 40 mg/day, 50 mg/day, and 60 mg/day) to estimate the percentage of the patients achieving a *trough* plasma level for the EC50 and EC90 SARS-CoV-2 inhibition. All analyses and graphing were conducted in R.

**Results. ** By day-10 at 20 mg/day 93.2% and 47% of the population will achieve the *trough* target plasma EC50 and EC90 concentrations, respectively, which translates to a lung tissue distribution coefficient of 60-times higher EC50 (283.6 ng/ml [0.82 mM]) and EC90 (1390.1 ng/ml [4.02 mM]). Further, by day-10 at an ideal dose of 40 mg/day, 99% and 93% of patients will reach the *trough* EC50 and EC90 concentrations, respectfully. Lastly, only a dose of 60 mg/day will reach the SARS-CoV-2 EC90 inhibitory concentration in the brain.

**Conclusion.** Overall, with a minimum treatment period of 10-days and a minimum dose of 20 mg/day, this study corroborates *in vitro* studies reporting fluoxetine inhibiting SARS-CoV-2 titers and also multiple *observational* clinical studies showing therapeutic benefit of fluoxetine in COVID-19 patients.

## Introduction

The selective serotonin reuptake inhibitor (SSRI) fluoxetine is a racemic mixture of two stereoisomers, *R*-fluoxetine and *S*-fluoxetine, and maintains regulatory approvals for a wide-array of clinical indications in the medical specialty of psychiatry. Several recent *in vitro* studies have shown that fluoxetine inhibits replication of the Severe Acute Respiratory Syndrome Coronavirus 2 (SARS-CoV-2) pathogen causing the worldwide novel coronavirus disease 2019 (COVID-19) pandemic ( Schloer *et al.*, 2020; Zimniak *et al.*, 2020, 2021; Dechaumes *et al.*, 2021; Fred *et al.*, 2021). The potential underlying mechanisms showing clinically protective factors in the SARS-CoV-2 infection is at least in-part due to fluoxetine being a functional inhibitor of acid sphingomyelinase (FIASMA) – the ceramide system – which is reported to play a critical central role in COVID-19, as shown in both preclinical ( Carpinteiro *et al.*, 2020a, 2021) and observational clinical studies ( Darquennes *et al.*, 2021; Hoertel *et al.*, 2021a, c). Further, another important mechanism is that fluoxetine (sigma-1 receptor, Ki=191.2 nM) and as fluvoxamine (sigma-1 receptor, Ki=17.0 nM), are endoplasmic reticulum-derived sigma-1 receptor agonists, which is known to inhibit cytokine production; thereby mitigating inflammation by decreasing tumor necrosis factor (TNF-α) and is protective against clinical deterioration in sepsis ( Roumestan *et al.*, 2007; Hashimoto, 2015; Köhler *et al.*, 2018; Rosen *et al.*, 2019; Sukhatme *et al.*, 2021). Similarly, Marín-Corral *et al*. evaluated 22 metabolomics biomarkers from plasma samples in hospitalized COVID-19 patients (n=49), in an effort to guide COVID-19 clinical decisions by disease severity, found that ceramide metabolism, tryptophan degradation, as well as reduction in nicotinamide adenine nucleotide reactions were significantly associated with respiratory severity and inflammation patients in COVID-19 patients ( Marín-Corral *et al.*, 2021). These known facts not only point to further understanding the pathogenesis and efficacy of fluoxetine in depression, but also is suggestive of the compound being repurposed in infectious diseases.

In preclinical studies, Zimniak *et al*. reported that following a three day incubation period of fluoxetine in Vero cells, inoculated at a multiplicity of infection (MOI) of 0.5, resulted in the median maximal effective concentration (EC50) of 387 ng/ml (1.1 μM) and further found a concentration of 800 ng/ml (2.3 μM) significantly inhibited SARS-CoV-2 replication ( Zimniak *et al*., 2020, 2021). Similarly, Schloer *et al*. found that fluoxetine significantly decreases SARS-Cov-2 titers, after a 48-hour incubation period, in both African green monkey kidney epithelial Vero E6 cells (EC50=0.69 μM and 90% maximal effective concentration [EC90]=1.81 μM, MOI=0.01) and human-lung Calu-3 cells (EC50=0.82 μM and EC90=4.02 μM, MOI=0.1) ( Schloer *et al*., 2020). Further, Dechaumes *et al.* reported that fluoxetine can inhibit SARS-CoV-2 replication in Vero E6 cells at a MOI of 0.01 reducing infectious titers below the limit of quantification after 48-hours at 10 μM ( Dechaumes *et al.*, 2021). Lastly, Fred *et al*., from the University of Helsinki, reported fluoxetine inhibits SARS-CoV-2 variants (B.1.1.7 and B.1.351) and the spike mutations (E484K, K417N, N501Y) in Calu-1 human lung epithelial cells at a median inhibitory concentration (IC50) of 5.992 μM ( Fred *et al*., 2021). Taken together these *in vitro* studies prove in a dose-dependent manner that the SSRI fluoxetine inhibits the SARS-CoV-2 pathogen known to cause the worldwide pandemic, the novel coronavirus disease 2019 (COVID-19).

Clinically, the fluoxetine SARS-CoV-2 *in vitro* findings were corroborated by Hoertel *et al*. who showed in a multicenter observational retrospective cohort study of patients treated with fluoxetine and diagnosed with COVID-19, experienced a lower risk of intubation and death (hazard ratio [HR]=0.32; 95% confidence interval [CI], 0.14–0.73, p=0.007) at a median fluoxetine dose of 20 mg (standard deviation [SD]=4.82) ( Hoertel *et al.*, 2020, 2021b). In addition, the association and/or effect of antidepressants improving clinical outcomes in COVID-19 have been confirmed in several recent observational clinical studies. Diez-Quevedo *et al*. aimed to identify how psychiatric disorders and psychopharmacological therapeutics prior to and throughout COVID-19 hospital admissions were related to mortality and found that out of 2,150 cases (between March 1, 2020 and November 17, 2020), 1,011 received psychotropics during admission (767 *de novo,* without history of psychotropics), and antidepressants (SSRI=220 [18.1%], mirtazapine=284 [59%]) were associated with a lower risk of mortality (HR=0.34, 95% CI, 0.17-0.67, p=0.002) ( Diez-Quevedo *et al.*, 2021). Hoertel *et al*. investigated compounds classified as FIASMAs, due to the ceramide/acid sphingomyelinase system being related to SARS-CoV-2 infection, found that out of a total of 2,846 hospitalized cases (277, 9.7% taking a FIASMA-based compounds), patients with FIASMA medications were significantly associated with lower likelihood of intubation or death (HR=0.71; 95% CI=0.58-0.87, p<0.001) ( Hoertel *et al.*, 2021c). Another study (n=545) by Hoertel *et al*. which also investigated FIASMA psychotropics against COVID-19, reported that in 164 (30.1%) patients who were treated with FIASMA-based compounds at baseline, had a significant lower risk of intubation or death (HR=0.42; 95%CI=0.31-0.57; p<0.01) ( Hoertel *et al.*, 2021a). Recently, Oskotsky and colleagues reported that among SSRIs, fluoxetine alone had a statistically significant lower relative risk of mortality in comparison to COVID-19 patients who were not prescribed the SSRI ( Oskotsky *et al.*, 2021). Nemeth *et al*. reported in a retrospective case-control study conducted at the Uzsoki Street Teaching Hospital at the Semmelweis University found that, when compared to patients not taking fluoxetine, patients taking fluoxetine (20 mg/day) and diagnosed with COVID-19 pneumonia was associated with a 70% decrease of mortality (odds-ratio = 0.33 [95% CI, 0.16–0.68, p=0.002]) ( Németh *et al.*, 2021). Most recently, in a large state psychiatric hospital operated by the New York State Office of Mental Health, Clelland *et al.* showed patients treated with fluoxetine (p=0.023) or trazodone (p = 0.001) had statistically significant lower risk of infection with COVID-19, while there was a trend of higher risk of infections with patients treated with the atypical antipsychotic olanzapine (p=0.084) ( Clelland *et al.*, 2022).

Two clinical trials showed another SSRI antidepressant fluvoxamine – also a FIASMA-based compound with sigma-1 receptor agonist properties – had clinical benefit against COVID-19 ( Lenze *et al.*, 2020; Seftel & Boulware, 2021). Lenze *et al*. reported in a double-blind randomized clinical trial (n=152) between April 10, 2020 and August 5, 2020, that none (0 of 80) of the patients who received fluvoxamine (n=80) at 100 mg/day compared to 8.3% (6 of 72) of patients receiving placebo (n = 72) three-times per day for 15 days experienced clinical deterioration (absolute difference=8.7%, 95% CI, 1.8%-16.4%, p=0.009) ( Lenze *et al.*, 2020). Seftel *et al*. found in a prospective cohort study, none (0 of 65) of the patients who received fluvoxamine 50 mg twice daily (100 mg/day) versus 12.5% (6 of 48) of patients who were observed alone were hospitalized and by day-14, residual COVID-19 symptoms were evident in none (0 of 65) of patients treated with fluvoxamine versus 60% (29 of 48) of patients who observed alone ( Seftel & Boulware, 2021).

Considering the COVID-19 clinical symptoms affecting the lungs, fluoxetine lung concentrations is a critically important factor to consider when interpreting study results of SARS-CoV-2 inhibition. Johnson *et al*. reported human-tissue concentrations of fluoxetine in airline pilots in whole-blood ranged from 0.021–1.4 μg/ml and lung concentrations ranged from 1.56 μg/ml to 51.9 μg/ml, leading to a fluoxetine distribution coefficient of 60, and is clinically relevant when investigating the pharmacokinetics of fluoxetine ( Johnson, Lewis & Angier, 2007). In this context, the aim of this study is to conduct *in silico* population pharmacokinetic dosing simulations, which serve as the pharmacometrics standard in clinical trials and regulatory drug application submissions, to quantify the percentage of patients expected to achieve the *trough* effective concentration resulting in 90% inhibition of SARS-CoV-2 as found in Calu-3 human lung cells.

## Methods

### Pharmacokinetic model

The pharmacometrics model that incorporates differential equation variables and the respective variances for a structural one-compartment pharmacokinetic model with first-order absorption was referenced from the United State Food and Drug Administration’s (FDA) Center for Drug Evaluation and Research Clinical Pharmacology and Biopharmaceutics Review(s) for a New Drug Application (NDA) 19-936 SE5-064 for Prozac (Fluoxetine Hydrochloride) that was submitted by Eli Lilly ( https://www.accessdata.fda.gov/drugsatfda_docs/nda/2003/18936S064_Prozac%20Pulvules_biopharmr.pdf) ( Center for Drug Evaluation and Research, 2002). For this study, the pharmacokinetic parameters referenced from the NDA were calculated by the FDA after combining 3 pharmacokinetic study datasets resulting in the following pharmacometric parameters: volume of distribution (Vd) value of 1,480 liters (variance [ω], 0.22), clearance rate (CL) value of 29.1 liters/hour (ω=0.376), and fixed absorption rate (Ka) of 0.67 (1/hour) (ω=not applicable as value was fixed) ( Center for Drug Evaluation and Research, 2002). As noted in the fluoxetine NDA, the influence of age was neither relevant for clearance nor volume of distribution ( Center for Drug Evaluation and Research, 2002).

*Target fluoxetine plasma concentration to achieve the EC50 and EC90 lung concentrations*

The molecular weight of fluoxetine hydrochloride is 345.8 g/mol and the reported EC50 (0.82 μM) and EC90 (4.02 μM) values from the Schloer *et al*. study are equivalent to EC50=283.6 ng/ml and EC90=1390.1 ng/ml, respectively. For all calculations, the *trough* target plasma concentrations will be referenced from the Schloer *et al*. study who reported after a 48-hour incubation period in Calu-3 lung cells ( Schloer *et al*., 2020) which are significantly higher than the EC90 in Vero E6 cell results from Zimniak *et al*. study ( Schloer *et al*., 2020; Zimniak *et al*., 2020, 2021).

### Dosing simulations

To estimate the percentage of patients from a population of one thousand simulated patients who would achieve the *trough* target EC90 concentration, pharmacokinetic dosing of fluoxetine consisted of three dosing trials of fluoxetine: 20 mg/day, 30 mg/day, 40 mg/day, 50 mg/day, and lastly 60 mg/day.

### Software and statistics

All pharmacokinetic dosing simulations are conducted with a population of 1,000 patients using *mrgsolve* and pharmacokinetic parameter estimates using *PKNCA* in R version 3.6.3 ( R Core Team, 2015). The overall R script has been adapted from a study published in Clinical Pharmacology and Therapeutics using hydroxychloroquine ( Al-Kofahi *et al*., 2020). Statistical results providing percentage estimates are calculated from *trough* concentrations of patients achieving the effective concentrations and is referenced from the Schloer *et al*. study reporting the EC50 and EC90 values in human-lung Calu-3 cells ( Schloer *et al*., 2020).

## Results

The fraction of fluoxetine bound in human plasma is 94%, which leaves only 6% of the compound being unbound in human plasma ( Sommi, Crismon & Bowden, 1987). Despite fluoxetine being highly protein bound, a study by Mantinieks *et al.* reported in paired antemortem and postmortem cases (n=18), fluoxetine concentrations had a human whole-blood to plasma ratio of 0.8-1.0, meaning that the *fluoxetine whole-blood concentrations are actually less than plasma or, at most, up to a 1:1 ratio* and would not require to be scaled from plasma to whole-blood ( Mantinieks *et al*., 2020). Moreover, Mantinieks *et al.* found the postmortem (range: 0.031–1.4 mg/L) to antemortem (range: 0.018–0.51 mg/L) fluoxetine drug concentration ratio as 1.8, but was not statistically significant as the p-value > 0.05 and thus the 1.8 ratio is not applicable to this study ( Mantinieks *et al*., 2020). Therefore, this study directly translated the simulated fluoxetine plasma concentrations and directly applied the tissue distribution coefficients (60 for lung, 15 for brain, 10 for heart, 38 for liver, 20 for spleen, and 9 for kidneys) from the Johnson *et al.* study and the original preprint version of this manuscript is updated to account for the findings from Mantinieks *et al.* ( Johnson, Lewis & Angier, 2007; Eugene, 2020, 2021; Mantinieks *et al*., 2020).

**Figure 1.**
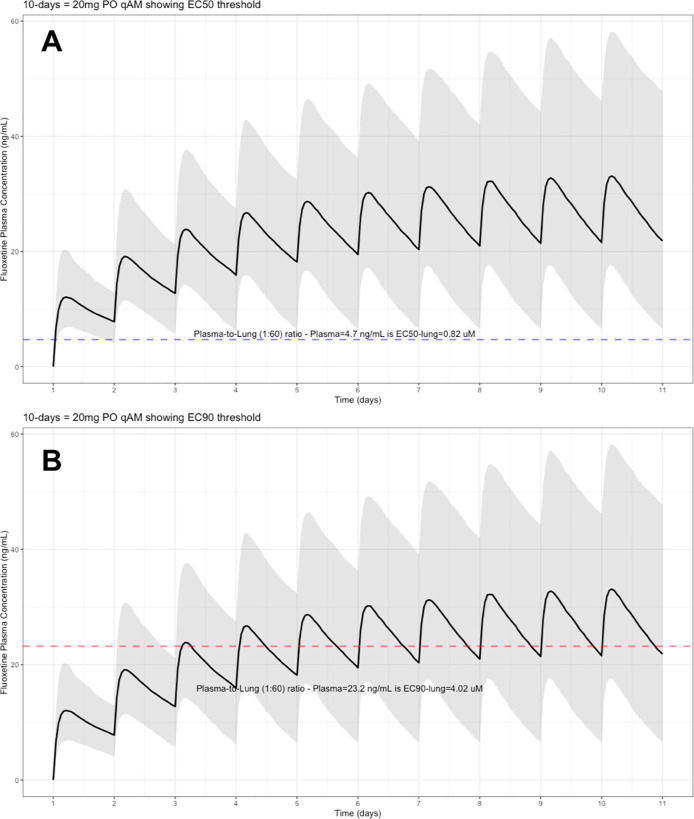
Fluoxetine population (n=1,000) dosing simulation results for an oral dose of *20 mg/day* for 10 days. The shaded regions illustrate the 10th (lower) and 90th (upper) percentiles with the solid line within the shaded region representing the median fluoxetine concentration. The dashed horizontal line depicts the effective concentration resulting (A) 50% and (B) 90% inhibition (EC90) of SARS-Cov-2 that will result in 60-times higher level in the lungs.

**Figure 2.**
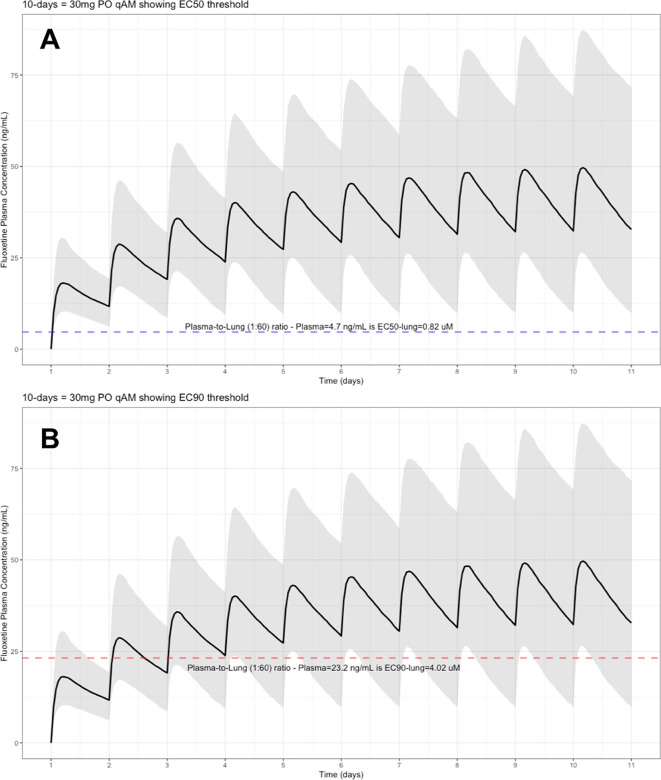
Fluoxetine population (n=1,000) dosing simulation results for an oral dose of *30 mg/day* for 10 days. The shaded regions illustrate the 10th (lower) and 90th (upper) percentiles with the solid line within the shaded region representing the median fluoxetine concentration. The dashed horizontal line depicts the effective concentration resulting (A) 50% and (B) 90% inhibition (EC90) of SARS-CoV-2 that will result in 60-times higher level in the lungs.

**Figure 3.**
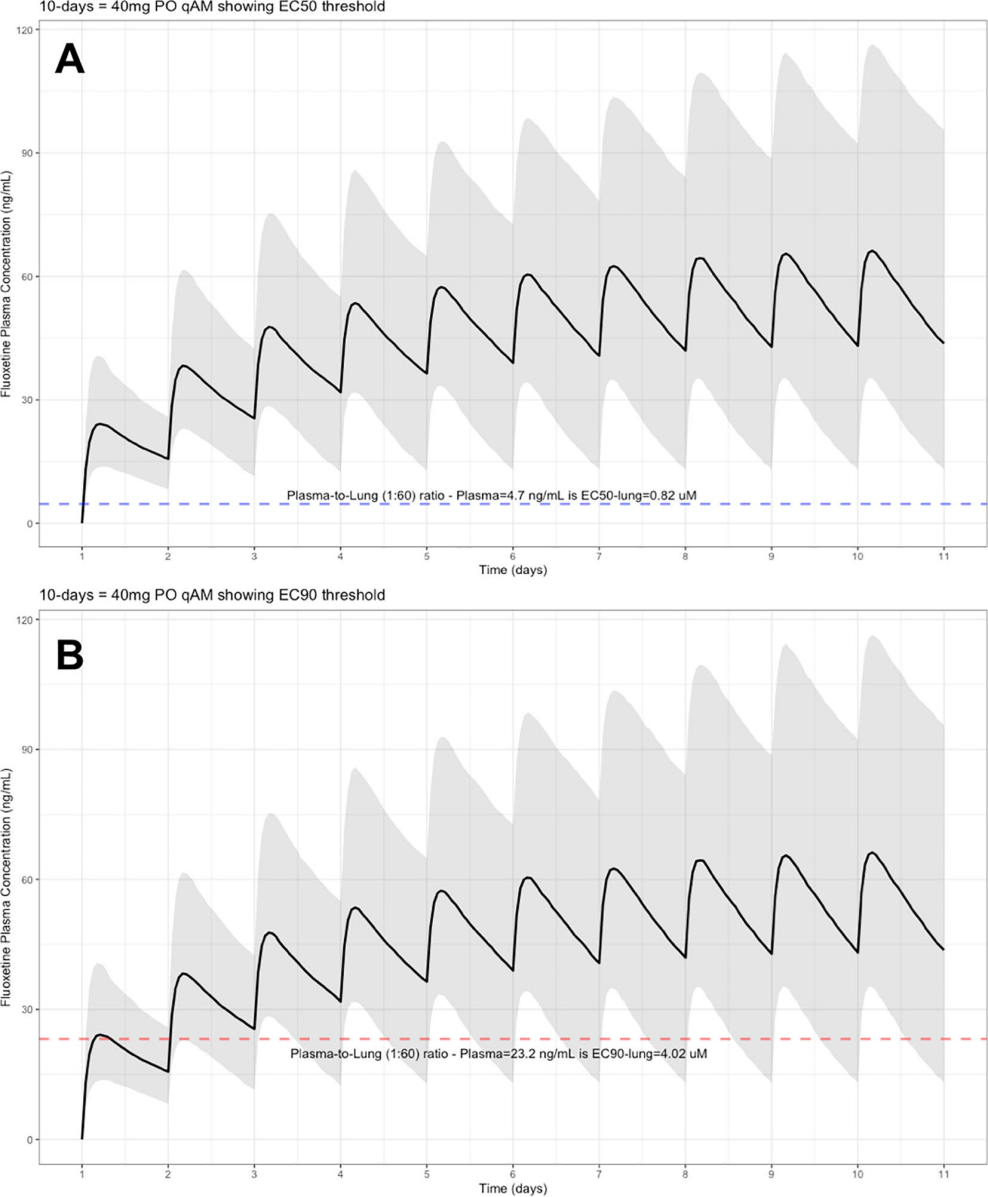
Fluoxetine population (n=1,000) dosing simulation results for an oral dose of *40 mg/day* for 10 days. The shaded regions illustrate the 10th (lower) and 90th (upper) percentiles with the solid line within the shaded region representing the median fluoxetine concentration. The dashed horizontal line depicts the effective concentration resulting (A) 50% and (B) 90% inhibition (EC90) of SARS-CoV-2 that will result in 60-times higher level in the lungs.

**Figure 4.**
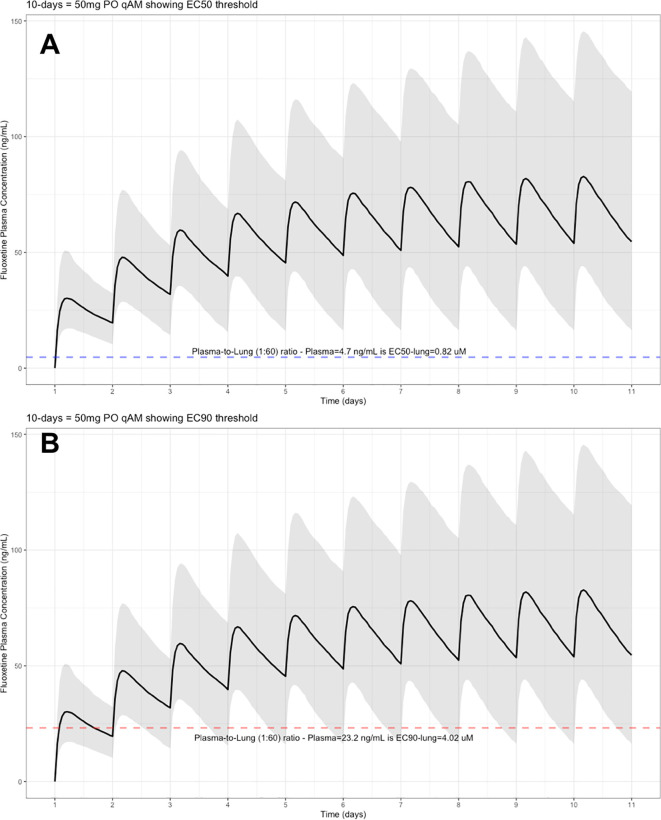
Fluoxetine population (n=1,000) dosing simulation results for an oral dose of *50 mg/day* for 10 days. The shaded regions illustrate the 10th (lower) and 90th (upper) percentiles with the solid line within the shaded region representing the median fluoxetine concentration. The dashed horizontal line depicts the effective concentration resulting (A) 50% and (B) 90% inhibition (EC90) of SARS-CoV-2 that will result in 60-times higher level in the lungs.

**Figure 5.**
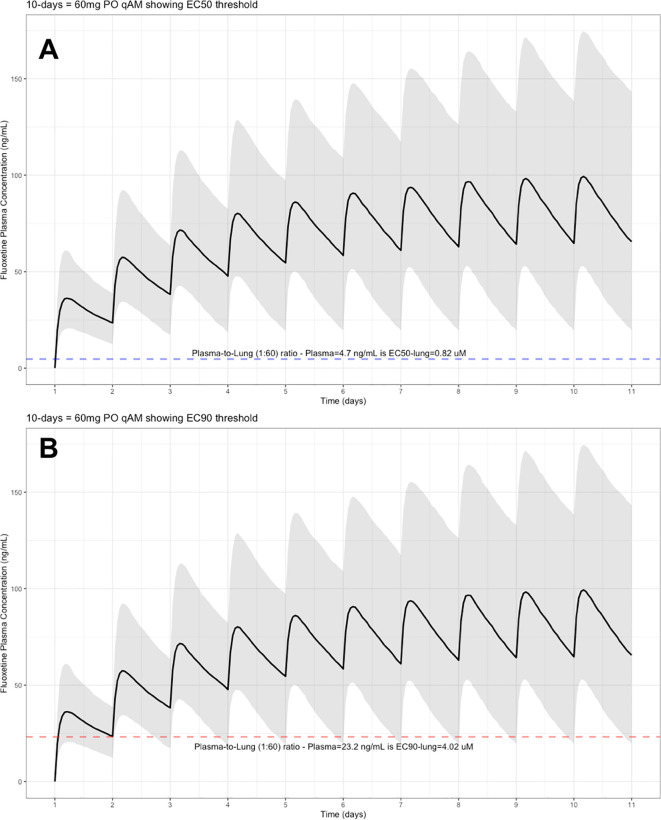
Fluoxetine population (n=1,000) dosing simulation results for an oral dose of *60 mg/day* for 10 days. The shaded regions illustrate the 10th (lower) and 90th (upper) percentiles with the solid line within the shaded region representing the median fluoxetine concentration. The dashed horizontal line depicts the effective concentration resulting (A) 50% and (B) 90% inhibition (EC90) of SARS-CoV-2 that will result in 60-times higher level in the lungs.

The target EC90 endpoint lung concentration for fluoxetine is 1390.1 ng/ml [4.02 μM] and 1/60 of this concentration is the new EC90-plasma concentration of 23.2 ng/ml [0.067 μM]. Similarly, the fluoxetine SARS-CoV-2 lung EC50 is 283.6 ng/ml [0.82 μM] and 1/60 of the EC50 at the target lung concentration in the plasma results in 4.7 ng/mL. The percentage of the 1,000 simulated patients are illustrated in the following figures: Figure 1 (20 mg/day), Figure 2 (30 mg/day), Figure 3 (40 mg/day), Figure 4 (50 mg/day), and Figure 5 (60 mg/day) with a horizontal dashed-line throughout the pharmacokinetic dosing figures showing the required *trough* EC50 and EC90-plasma levels that translates to the 60-times higher lung concentrations.

At the peak fluoxetine concentration (Cmax), the corresponding median time Tmax is 220 hours (range, 49–220) and has a half-life (t ½) – expressed as arithmetic mean (standard deviation, SD) – of 46.6 hours (SD=40.8) for the study population. Further, in a population of 1,000 patients during a fluoxetine treatment period of once daily dosing for 10-days, the following are the maximum lung concentrations (Cmax-lungs): 20 mg/day (Cmax-lungs = 1950 ng/mL [5.6 μM]), 30 mg/day (Cmax-lungs=2922 ng/mL [8.4 μM]), 40 mg/day (Cmax-lungs=3900 ng/mL [11.3 μM]), 50 mg/day (Cmax-lungs=4872 ng/mL [14.1 μM], 60 mg/day (Cmax=5844 ng/mL [16.9 μM]). At all doses ranging from 20 mg/day to 60 mg/day, oral fluoxetine doses were able to exceed the target SARS-CoV-2 EC50 [0.82 μM] and EC90 [4.02 μM] inhibitory values. All fluoxetine doses of at least 20 mg/day exceeds the EC50 in the brain; however, only a fluoxetine dose of 60 mg/day exceeds the EC90 in the brain resulting in 90% inhibition of the SARS-CoV-2 titers and likely most beneficial for COVID-19 and likely treatment-resistant depression associated with inflammation. Detailed results of all of the maximum concentrations in the plasma, whole-blood, lungs, heart, liver, spleen, and kidney are found in Table 1. Of note, only a fluoxetine dose of 60 mg/day will achieve the EC90 concentration within the brain and may likely be most effective in neuropsychiatric symptoms of COVID-19.

**Table 1.** Fluoxetine pharmacokinetics showing organ concentrations (ng/mL and μM) aiming to achieve the target SARS-CoV-2 EC50 (283.6 ng/ml [0.82 μM]) and EC90 (1390.1 ng/ml [4.02 μM]) by day-10 of treatment in humans.

Organ	Fluoxetine organ distribution concentrations at standard antidepressant doses				
	20 mg/day	30 mg/day	40 mg/day	50 mg/day	60 mg/day
	Cmax ng/ml [μM]	Cmax ng/ml [μM]	Cmax ng/ml [μM]	Cmax ng/ml [μM]	Cmax ng/ml [μM]
Plasma	32.5 [0.09]	48.7 [0.14]	65 [0.19]	81.2 [0.23]	97.4 [0.28]
Whole-Blood	32.5 [0.09]	48.7 [0.14]	65 [0.19]	81.2 [0.23]	97.4 [0.28]
Lung	1950 [5.6]	2922 [8.4]	3900 [11.3]	4872 [14.1]	5844 [16.9]
Brain	487.5 [1.4]	730.5 [2.1]	975 [2.8]	1218 [3.5]	1461 [4.2]
Heart	325 [0.9]	487 [1.4]	650 [1.9]	812 [2.3]	974 [2.8]
Liver	1235 [3.6]	1850.6 [5.4]	2470 [7.1]	3085.6 [8.9]	3701.2 [10.7]
Spleen	650 [1.9]	974 [2.8]	1300 [3.8]	1624 [4.7]	1948 [5.6]
Kidney	292.5 [0.8]	438.3 [1.3]	585 [1.7]	730.8 [2.1]	876.6 [2.5]

Maximum plasma concentration (Cmax) are expressed as geometric mean (geometric coefficient of variation, CV%). Fluoxetine tissue distribution coefficients (60 for lung, 15 for brain, 10 for heart, 38 for liver, 20 for spleen, and 9 for kidneys) as reported ( Johnson, Lewis & Angier, 2007).

These aforementioned pharmacokinetic results translate to at the end of day-1, a dose of 20 mg/day, 85.7% and only 0.3% of the population reaching the target EC50 and EC90 concentrations in the lungs at the end of Day-1 of fluoxetine treatment, respectively. Whereas, at a dose of 60 mg/day for Day-1, 98.7% achieves EC50 and 51.6% reaches the EC90 targets for the lungs. The time to achieving these target EC50 and EC90 lung concentrations are assumed to be equivalent to that in whole-blood due to the high physiological blood flow rate into the lungs. Table 2 provides all of the pharmacodynamic population outcomes of the percentage of the patient population achieving the EC50 and EC90 *trough* target lung concentrations.

**Table 2.** Predicted SARS-CoV-2 pharmacodynamics showing the daily fluoxetine dose and corresponding percent of the population (n=1,000) achieving a *trough* plasma concentration of 4.7 ng/ml (EC50-plasma) and 23.2 ng/ml (EC90-plasma), which leads to 60-times higher lungs based on the target EC50 (283.6 ng/ml [0.82 μM]) and EC90 (1390.1 ng/ml [4.02 μM]) concentrations, during a treatment period of 10-days.

	20 mg/day		30 mg/day		40 mg/day		50 mg/day		60 mg/day	
Time point	%EC50	%EC90	%EC50	%EC90	%EC50	%EC90	%EC50	%EC90	%EC50	%EC90
Day 1	85.7	0.3	94.9	4.2	97	14.7	98.2	34	98.7	51.6
Day 2	93	6.7	96.4	33.3	97.6	56.9	98.6	72.4	98.7	82.1
Day 3	93.2	19.4	96.5	52.6	97.6	71.2	98.6	81.5	98.7	85.9
Day 4	93.2	30.8	96.6	59.7	97.7	75.4	98.6	83	98.7	87.1
Day 5	93.2	37.9	96.6	64.2	97.7	77.6	98.6	83.7	98.7	87.4
Day 6	93.2	42.3	96.6	66.1	97.8	78.1	98.6	83.9	98.7	87.4
Day 7	93.2	44.5	96.6	67.1	97.8	78.5	98.6	83.9	98.7	87.6
Day 8	93.2	45.8	96.6	67.6	97.8	78.5	98.6	83.9	98.7	87.6
Day 9	93.2	46.6	96.6	67.8	97.8	78.5	98.6	83.9	98.7	87.6
Day 10	93.2	47	96.6	68	97.8	78.6	98.6	83.9	98.7	87.6

**Figure 6.**
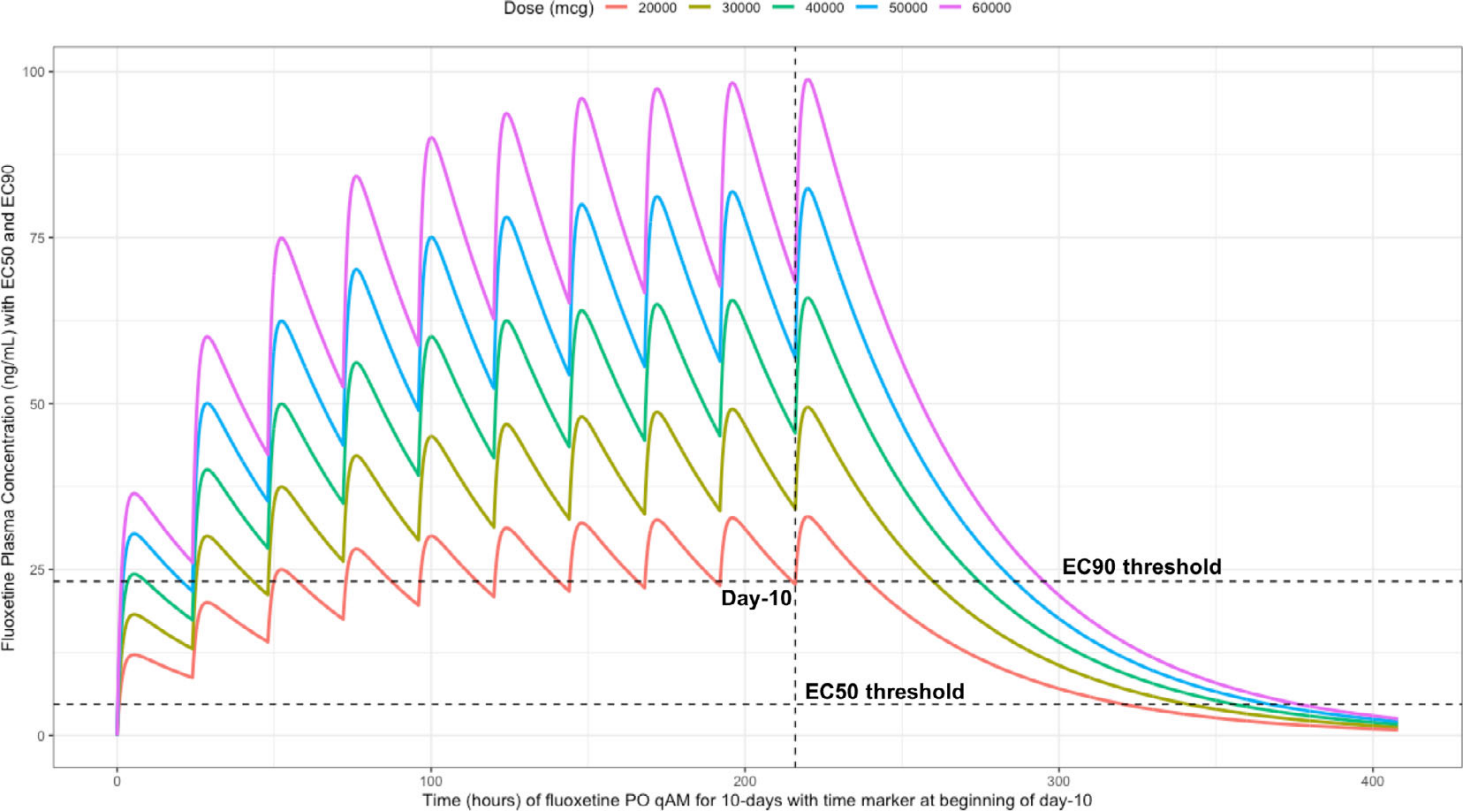
Fluoxetine concentration-time profile in human plasma at standard daily antidepressant doses (20 mg/day, 30 mg/day, 40mg/day, 50 mg/day, 60 mg/day) throughout 10 days. The vertical line illustrates the morning dose on day-10, the highest (EC90) and lowest (EC50) horizontal lines illustrates the threshold for the plasma effective concentrations inhibiting 50% and 90% of SARS-CoV-2 titers that distributes to 60-times higher concentrations within the lungs, respectively.

**Figure 7.**
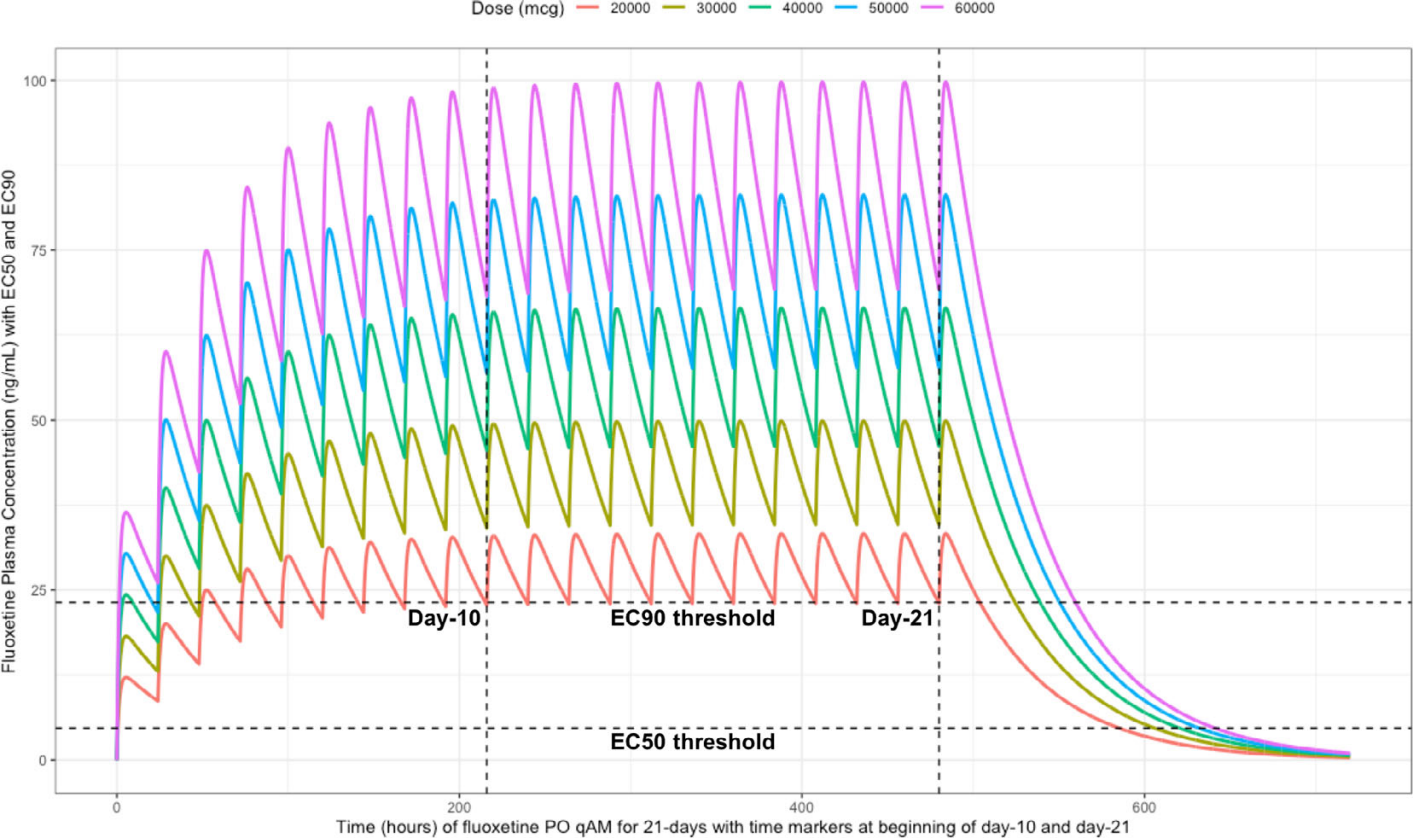
Fluoxetine concentration-time profile in human plasma at standard daily antidepressant doses (20 mg/day, 30 mg/day, 40mg/day, 50 mg/day, 60 mg/day) throughout 21 days. The two vertical line illustrates the morning doses on day-10 and on day-21. The highest (EC90) and lowest (EC50) horizontal lines illustrates the threshold for the plasma effective concentrations inhibiting 50% and 90% of SARS-CoV-2 titers that distributes to 60-times higher concentrations within the lungs, respectively.

Using the mean fluoxetine pharmacokinetic dosing parameters, as graphically depicted in Figure 6 and Figure 7, the *trough* plasma (Cmin) and lung concentrations, prior to the morning dose on Day-10, are identified to be: Cmin _20mg_=22.8 ng/ml (lung=1368 ng/ml [4.0 μM]), Cmin _30mg_=34.1 ng/ml (lung=2046 ng/ml [5.9 μM]), Cmin _40mg_=45.5 ng/ml (lung= 2730 ng/ml [7.9 μM]), Cmin _50mg_=56.9 ng/ml (lung=3414 ng/ml [9.9 μM]), and Cmin _60mg_=68.3 ng/ml (lung= 4098 ng/ml [11.9 μM]).

The difference between the Cmax and Cmin in this study is approximately 20 ng/ml and to calculate the equivalent micromolar concentration, the ng/ml value is divided by the molecular weight of 345.8 g/mol. With a t ½ of 46.6 hours and summing the t ½ with the standard deviation of 40.8 hours, a portion of the population will take 21-days to reach steady-state. Further, assuming all patients are treated with fluoxetine for either 10-days or 21-days and with the last dose being on the morning of either day-10 or day-21, the amount of time it would take for the plasma concentration to fall beneath the threshold plasma EC50 (4.7 ng/ml [0.014 uM]) and EC90 (23.2 ng/ml [0.067 μM]) values that results in 60-times higher lungs (EC50=283.6 ng/ml [0.82 μM]) and EC90=1390.1 ng/ml [4.02 μM]), are as follows: 20 mg/day (3.4 days to EC50; 0 days to EC90), 30 mg/day (4.2 days to EC50; 0.8 days to EC90), 40 mg/day (4.8 days to EC50; 1.5 days to EC90), 50 mg/day (5.3 days to EC50; 1.9 days to EC90), and 60 mg/day (5.7 days to EC50; 2.3 days to EC90). Figure 6 illustrates the fluoxetine pharmacokinetics over 10-days and Figure 7 shows fluoxetine pharmacokinetics throughout 21-days for once daily dosing in the morning with EC50 and EC90 threshold values.

## Discussion

According to the United States Food and Drug Administration Adverse Events Reporting System (FAERS) during the window period of 1982 to June 30, 2020, fluoxetine was reported to have a total of 79,929 cases, 62,948 serious cases, and 10,043 end of life cases ( https://www.fda.gov/drugs/questions-and-answers-fdas-adverse-event-reporting-system-faers/fda-adverse-event-reporting-system-faers-public-dashboard). Females represented 58% of the adverse drug reactions (ADRs), males represented 27% of the ADRs, and 15% of the ADRs did not specify a gender. The most common adverse drug event reported for fluoxetine is Drug Interaction and amounts to 3,798 cases (4.75% of total). Given this information, drug interactions associated with fluoxetine are due to inhibition of the cytochrome P450 (CYP) system. Specifically, CYP2C19 and CYP2D6 may have interactions such as in patients taking tamoxifen for breast cancer by inhibiting conversion to the active endoxifen metabolite via CYP2D6 or in cases of clopidogrel in cardiology by inhibiting the conversion of clopidogrel to the active 2-oxo-clopidogrel metabolite ( Spina, Trifirò & Caraci, 2012; Eugene, 2019).

Extrapolating from *in vitro* to *in vivo* concentrations are dependent on intracellular versus extracellular concentrations, as well as the methodology of quantifying either whole-blood versus plasma concentrations in human pharmacokinetic studies. The EC50 and EC90 target concentrations represent the extracellular fluoxetine concentrations in the SARS-CoV-2 cell culture media. As COVID-19 is known to affect the brain during active infection and in post-COVID-19 states, adequate brain concentrations would be clinically important in patients who may experience depression. Bolo *et al*. reported fluoxetine brain concentrations, at steady-state, using fluorine magnetic spectroscopy and showed fluoxetine concentrations were 10-times higher in the brain than in human plasma ( Bolo *et al*., 2000). Specifically, Bolo *et al*. found in study participants taking oral doses (10 mg, n=1; 20 mg, n=1; 40 mg, n=2) with a treatment period ranging from three months to 12-months that fluoxetine human brain concentrations were 13 μM (SD=7) versus 1.73 μM (SD=1.00) in human plasma fluoxetine ( Bolo *et al*., 2000). In comparison, Johnson *et al*. found the coefficients for tissue distribution of fluoxetine relative to whole-blood was: 60× higher for the lung, 15× for brain, 10× for heart, 38× for liver, 20× for spleen, and 9× higher for the kidneys ( Johnson, Lewis & Angier, 2007).

As patients recover from the acute COVID-19 symptoms, long-term sequelae are being documented and in one of the long COVID-19 study in young patients reported that 92% were found to have ongoing cardiorespiratory symptoms with organ dysfunction and impairment in the lungs (33%), heart (32%), kidneys (12%) ( Dennis *et al*., 2020). In another long COVID-19 syndrome study, 96% of the patients were female and experienced statistically significant exercise intolerance, dyspnea, and chest pain when compared to those not diagnosed with COVID-19 ( Walsh-Messinger *et al*., 2020). Moreover, Walsh-Messinger *et al*. found patients with long COVID-19 syndrome had higher ratings of depression subscale markers of altered sleep and thinking, but depression severity was not significantly different with patients not diagnosed with COVID-19 ( Walsh-Messinger *et al*., 2020). As shown in Table 1, a fluoxetine dose of 60 mg/day achieves the EC90 concentration in the brain and would likely benefit patients with neuropsychiatric symptoms of COVID-19. In addition to *fluoxetine*, other psychotropics ( *fluvoxamine, hydroxyzine,* and *trazodone*) and one antihypertensive ( *amlodipin*e) are reported to be associated with reducing risk of death in patients with COVID-19 ( Zhang *et al.*, 2020; Darquennes *et al.*, 2021; Sánchez-Rico *et al.*, 2021; Clelland *et al.*, 2022; Reis *et al.*, 2022). Further, Reznikov *et al*. showed three antihistamines ( *azelastine, diphenhydramine, and hydroxyzine*) have direct inhibitory activity against SARS-CoV-2 *in vitro;* while clinically, Reznikov *et al*. showed azelastine, cetirizine, diphenhydramine, hydroxyzine, and loratadine was significantly associated with a lower incidence of testing positive for COVID-19 in patients 61-years-old and older, but only cetirizine had the same association in patients 31-years-old and older ( Reznikov *et al.*, 2020).

Direct clinical translation of this current pharmacokinetic study supports the findings from a retrospective multicenter observational study by Hoertel *et al*., who found a median fluoxetine dose of 20 mg/day resulted in a significantly lower risk of intubation and death in a population composed of 63% women and 37% men ( Hoertel *et al.*, 2020, 2021b). Comparing the Hoertel *et al*. and Zimniak *et al*. publications, Hoertel *et al*. found that in addition to fluoxetine, venlafaxine (median dose of 75 mg/day) and escitalopram (median dose of 10 mg) were also associated with a lower risk of intubation and death; however, Zimniak *et al*. showed that neither escitalopram nor paroxetine inhibited SARS-CoV-2 *in vitro* ( Hoertel *et al.*, 2020, 2021b; Zimniak *et al.*, 2020, 2021). There have been some who have suggested that the effect of fluoxetine in treating depression is due to a placebo effect; however, with all due respect, it suggests of the lack of knowledge of the etiologies of depression and clinical pharmacology of fluoxetine beyond serotonin transporters to inhibiting inflammatory cytokines and more ( Kirsch & Sapirstein, 2004).

Antiviral properties of fluoxetine are well reported in the biomedical literature. Carpinteiro *et al*. reported that fluoxetine inhibits acid sphingomyelinase preventing infection of both cultured cells and human nasal epithelial cells in SARS-CoV-2, as well as in vesicular stomatitis virus pseudoviral particles presenting the SARS-CoV-2 spike protein ( Carpinteiro *et al.*, 2020b). A study by Zuo *et al*. showed fluoxetine resulted in potent inhibition of the coxsackievirus by reducing both synthesis of viral RNA and protein (EC50 of 2.3 μM or 795.34 ng/ml) exhibiting peak antiviral properties at 6.25 μM (2161.25 ng/ml) ( Zuo *et al*., 2012). Bauer *et al*. showed, in a broad-spectrum manner, fluoxetine inhibited enterovirus (picornaviridae family) replication with the *S*-fluoxetine enantiomer exhibiting a 5-fold lower EC50 than the racemic mixture of *R-* and *S*-fluoxetine ( Bauer *et al*., 2019). Further, Bauer *et al*. found the following fluoxetine EC50 values for the following pathogens: coxsackievirus B3 (racemate-EC50=2.02 μM or 698.5 ng/ml, *S*-fluoxetine-EC50=0.42 μM or 145.2 ng/ml), enterovirus EV-D68 (racemate-EC50=1.85 μM or 639.7 ng/ml, *S*-fluoxetine-EC50=0.67 μM or 231.7 ng/ml), and *S*-fluoxetine values alone for rhinovirus HRV-A2 (EC50=7.95 μM or 2749.1 mg/ml) and HRV-B14 (EC50=6.34 μM or 2192.4 ng/ml) ( Bauer *et al*., 2019). Notably, Zimniak *et al*. found that individual stereoisomers, *R*-fluoxetine and *S*-fluoxetine, inhibited the SARS-CoV-2 viral load; however, in contrast, fluoxetine could not inhibit gene expression of the herpes simplex-1 virus, human herpes virus-8, rabies virus, nor the respiratory syncytial virus ( Zimniak *et al*., 2020, 2021). As shown in Table 1, standard fluoxetine doses are capable of achieving the aforementioned EC50s for all of the aforementioned microbes and fluoxetine may be combined with antipsychotics (e.g. olanzapine) to treat bipolar depression, treatment-resistant depression, schizophrenia in general and these antipsychotics may have notable associations with sedation and somnolence ( Eugene *et al.*, 2021).

Optimal dose selection for clinical trials are of importance to ensure the maximum number of patients achieve the effective concentration resulting in 90% inhibition of the SARS-CoV-2 pathogen similarly to that reported in Calu-3 human lung cells. Based on the study findings showing the percentage of the population achieving the EC50 and EC90 inhibitory concentrations in the lungs, a dose of 40 mg/day would be recommended to achieve therapeutic potential inhibiting SARS-CoV-2 titers. Overall, given the abundance of clinical trial data and published clinical studies investigating various dosing approaches (e.g. loading dose) for fluoxetine, this author defers all final dosing recommendations for fluoxetine to the FDA approved package-insert for adults and for the pediatrics population (Eli Lilly / Dista Products Company, 2021). For an application in pediatrics, fluoxetine doses in children are recommended to start at 10 mg/day and then titrate to 20 mg/day, whereas in adults with depression, a 20 mg/day is recommended as a starting dose ( Eli Lilly/Dista Products Company, 2021). Lastly, given pharmacogenomic (drug-gene), DDIs, and drug-drug-gene interactions that are likely in patients taking multiple medications, therapeutic drug monitoring of fluoxetine and other co-administered medications are recommended.

A limitation of this study is that the results are purely quantitative using pharmacometric simulations based on differential equations and not a randomized controlled clinical trial. Despite this limitation, it is important for the reader to note that the methodology used in this study is standard practice for all drugs undergoing Phase-1 Clinical Trials in drug development by clinical pharmacologists for later regulatory approval for human use. Moreover, the pharmacometric parameters used were estimated from an NDA submitted to the United States Food and Drug Administration with parameter estimates actually calculated by the FDA clinical pharmacologists as reported in the Clinical Pharmacology and Biopharmaceutics Review after combining three human study datasets provided by the study sponsor ( https://www.accessdata.fda.gov/drugsatfda_docs/nda/2003/18936S064_Prozac%20Pulvules_biopharmr.pdf). Overall, from a drug-safety perspective, prior to administering fluoxetine, a careful review of all current medications and clinical status by a physician clinical pharmacologists to avoid drug interactions due to fluoxetine’s ability to strongly inhibit CYP2C19 and CYP2D6 ( Hefner, 2018). Compounds that are sensitive and moderate CYP2C19 substrates (e.g. omeprazole, diazepam, lansoprazole, rabeprazole, voriconazole) and CYP2D6 substrates (e.g. dextromethorphan, eliglustat, nebivolol, tolterodine, encainide, metoprolol, propranolol, tramadol) will have an increased total area under the concentration-time curve of ≥ 5-fold drug exposure when treated with fluoxetine ( https://www.fda.gov/drugs/drug-interactions-labeling/drug-development-and-drug-interactions-table-substrates-inhibitors-and-inducers). Lastly, patients who have a pharmacogenomic profile of being a CYP2D6 Ultra-rapid Metabolizers may have sub-therapeutic fluoxetine concentrations; while, CYP2D6 Poor Metabolizer or Intermediate Metabolizers may have supratherapeutic concentrations and should be monitored for potential fluoxetine side-effects; but may also have a higher rate of achieving the target *trough* EC90 concentration at a 20 mg/day fluoxetine dose relative to CYP2D6 Normal (Extensive) Metabolizers.

## Conclusions

This study investigated fluoxetine pharmacokinetics and human organ distribution which confirmed that previously published median effective concentrations and specifically, the EC90 fluoxetine value inhibiting SARS-CoV-2 in Calu-3 human lung cells are achievable using standard fluoxetine antidepressant doses (20 mg/day, 30 mg/day, 40 mg/day, 50 mg/day, and 60 mg/day) and also corroborates findings from multiple retrospective clinical studies showing patients who were exposed to fluoxetine during COVID-19 were associated with reduced risk of clinical deterioration and death. Overall, assuming patients are not treated with medications that result in significant DDIs or have the clinically relevant pharmacogenomic concerns, a minimum dose of 20 mg/day for at least 10-days inhibits SARS-CoV-2 viral titers due to efficient lung distribution at a 60-times higher concentration. Lastly, a dose of 60 mg/day achieves the SARS-CoV-2 EC90 concentration within the human brain and provides further insight to utility of pharmacokinetics and pharmacodynamics modeling and simulation to advancing medical sciences, recommending doses for clinical trials, and advancing patient care.

## Data availability

### Underlying data

*Open Science Framework*: All pharmacokinetic data presented in this study are available for download at the following link: https://doi.org/10.17605/OSF.IO/RVYPZ.

This project contains the following underlying data:

• Data File 1: v2_fluoxetine_20mg_PO_QAM.csv
• Data File 2: v2_fluoxetine_30mg_PO_QAM.csv
• Data File 3: v2_fluoxetine_40mg_PO_QAM.csv
• Data File 4: v2_fluoxetine_50mg_PO_QAM.csv
• Data File 5: v2_fluoxetine_60mg_PO_QAM.csv
• Data File 6: v2_fluoxetine_all_doses_10days.csv
• Data File 7: v2_fluoxetine_all_doses_21days.csv

### Software availability

Open Science Framework: The R programming language pharmacokinetic script that produces the general results of this study are available for download at: https://doi.org/10.17605/OSF.IO/RVYPZ ( Eugene, 2021).

This project contains the following software:

• v2_Fluoxetine_pharmacokinetic_sars_cov2_simulation_script - publication - DrAndy_R_EugeneMDPhD.R

Data are available under the terms of the Creative Commons Zero “No rights reserved” data waiver (CC0 1.0 Public domain dedication).
