# Supplementary material for: Fluoxetine pharmacokinetics and tissue distribution quantitatively supports a therapeutic role in COVID-19 at a minimum dose of 20 mg per day
Source: F1000Res. 2022 Oct 13;10:477. Originally published 2021 Jun 16. [Version 3] doi: 10.12688/f1000research.53275.3 (PMC9561539; doi:10.12688/f1000research.53275.3)
Supplement: Peer review history

# Peer review: Reviewer response for version 3

**DOI:** 10.5256/f1000research.136846.r153198

**Published:** 2022-10-13

**Authors:** Eero Castren

**Affiliations:** 1 Neuroscience Center, Helsinki Institute of Life Science HiLIFE, University of Helsinki, Helsinki, Finland

The revised article is fine.

Is the work clearly and accurately presented and does it cite the current literature?

Yes

If applicable, is the statistical analysis and its interpretation appropriate?

Yes

Are all the source data underlying the results available to ensure full reproducibility?

Yes

Is the study design appropriate and is the work technically sound?

Yes

Are the conclusions drawn adequately supported by the results?

Partly

Are sufficient details of methods and analysis provided to allow replication by others?

Yes

Reviewer Expertise:

Neuropharmacology

I confirm that I have read this submission and believe that I have an appropriate level of expertise to confirm that it is of an acceptable scientific standard.

# Peer review: Reviewer response for version 2

**DOI:** 10.5256/f1000research.119937.r121956

**Published:** 2022-02-25

**Authors:** Eero Castren

**Affiliations:** 1 Neuroscience Center, Helsinki Institute of Life Science HiLIFE, University of Helsinki, Helsinki, Finland

The author has only partially responded to the comments I gave to the previous version. It is also very difficult to see what changes have actually been made to the version 1.

Is the work clearly and accurately presented and does it cite the current literature?

Yes

If applicable, is the statistical analysis and its interpretation appropriate?

Yes

Are all the source data underlying the results available to ensure full reproducibility?

Yes

Is the study design appropriate and is the work technically sound?

Yes

Are the conclusions drawn adequately supported by the results?

Partly

Are sufficient details of methods and analysis provided to allow replication by others?

Yes

Reviewer Expertise:

Neuropharmacology

I confirm that I have read this submission and believe that I have an appropriate level of expertise to confirm that it is of an acceptable scientific standard, however I have significant reservations, as outlined above.

# Peer review: Reviewer response for version 2

**DOI:** 10.5256/f1000research.119937.r121955

**Published:** 2022-02-09

**Authors:** Nicolas Hoertel

**Affiliations:** 1 Hôpital Corentin-Celton, Service de Psychiatrie, Assistance Publique-Hôpitaux de Paris (AP-HP), Issy-les-Moulineaux, France; 2 Paris University, Paris, France

All my comments have been well addressed. I thank and congratulate the author for this very important PK modelling study.

Is the work clearly and accurately presented and does it cite the current literature?

Yes

If applicable, is the statistical analysis and its interpretation appropriate?

Yes

Are all the source data underlying the results available to ensure full reproducibility?

Yes

Is the study design appropriate and is the work technically sound?

Yes

Are the conclusions drawn adequately supported by the results?

Yes

Are sufficient details of methods and analysis provided to allow replication by others?

Yes

Reviewer Expertise:

Psychiatry; antidepressants; methodology; biostatistics; epidemiology; sphingomyelinase; COVID-19.

I confirm that I have read this submission and believe that I have an appropriate level of expertise to confirm that it is of an acceptable scientific standard.

# Peer review: Reviewer response for version 1

**DOI:** 10.5256/f1000research.56641.r87690

**Published:** 2021-06-23

**Authors:** Eero Castren

**Affiliations:** 1 Neuroscience Center, Helsinki Institute of Life Science HiLIFE, University of Helsinki, Helsinki, Finland

Andy Eugene has written an interesting paper on a very timely topic.  Preclinical as well as limited clinical data support the idea that some antidepressants and related compounds may protect cells from SARS-CoV2 infection, but it has been unclear whether concentrations that are found in cell culture studies to be inhibitory are really achieved in clinical treatments.  It is unfortunate that there is only limited amount of high-quality data on the tissue concentrations and distribution of fluoxetine in human tissues, but Eugene has taken advantage of the data that is available to model fluoxetine pharmacokinetics in human lung tissue.  His results suggest that sufficiently high lung tissue concentrations are achieved within a 10 day treatment period in majority of patients with a standard 20 mg/day dosing, and with higher doses, the trough concentration is achieved within the first days of treatment.  The simulations appear properly conducted and the manuscript is well written.  I have a few issues that might improve the manuscript further. 

  

1. The conclusions about how fast trough concentrations are achieved is based on the assumption that fluoxetine immediately distributes to lung tissue at the estimated 60 fold concentration compared to plasma levels.  However, there is some (although clearly incomplete) information that fluoxetine may accumulate into tissues over time. Rasenick and colleagues have found that certain antidepressants accumulate in lipid rafts in vitro over several days of exposure (Erb *et al. *2016 ^1^) For human brain, Karson *et al.* have found that, using spectroscopy, fluoxetine/norfluoxetine is not detectable in human brain at about 1 week, but at about 2-3 weeks of treatment, concentrations of several micromolar can be assayed (Karson *et al. *(1993 ^2^))  indicating that fluoxetine accumulates in brain over time.  If the same is true for lung tissue, the 60-fold concentrations may not be reached in lung tissues as quickly as estimated here.  Although paucity of information prevents any proper estimations, I think it would be important to discuss this issue in the discussion section.
2. The author should be complemented for indicating in many places in the manuscript fluoxetine concentrations in both ng/ml and in molar units, however, this is not done systematically. It would be very helpful to, wherever possible, indicate both units. In the figures, it may not be feasible to have double set of units in the Y-axis, I would prefer molar units, but if the author prefers ng/ml, that is not a major problem.
3. The author may want to mention that a clinical phase 3 trial evaluating the effect of fluvoxamine vs. placebo is ongoing and should be completed by September this year.

Is the work clearly and accurately presented and does it cite the current literature?

Yes

If applicable, is the statistical analysis and its interpretation appropriate?

Yes

Are all the source data underlying the results available to ensure full reproducibility?

Yes

Is the study design appropriate and is the work technically sound?

Yes

Are the conclusions drawn adequately supported by the results?

Partly

Are sufficient details of methods and analysis provided to allow replication by others?

Yes

Reviewer Expertise:

Neuropharmacology

I confirm that I have read this submission and believe that I have an appropriate level of expertise to confirm that it is of an acceptable scientific standard, however I have significant reservations, as outlined above.

## References

[1] : Antidepressants Accumulate in Lipid Rafts Independent of Monoamine Transporters to Modulate Redistribution of the G Protein, Gαs. *Journal of Biological Chemistry* .2016;291(38) : 10.1074/jbc.M116.727263 19725-19733 10.1074/jbc.M116.727263 PMC502566427432886

[2] : Human brain fluoxetine concentrations. *J Neuropsychiatry Clin Neurosci* .1993;5(3) : 10.1176/jnp.5.3.322 322-9 10.1176/jnp.5.3.322 8369643

# Peer review: Reviewer response for version 1

**DOI:** 10.5256/f1000research.56641.r87881

**Published:** 2021-06-21

**Authors:** Nicolas Hoertel

**Affiliations:** 1 Hôpital Corentin-Celton, Service de Psychiatrie, Assistance Publique-Hôpitaux de Paris (AP-HP), Issy-les-Moulineaux, France; 2 Paris University, Paris, France

This is a review of the manuscript "Fluoxetine pharmacokinetics and tissue distribution suggest a possible role in reducing SARS-CoV-2 titers" submitted for publication in *F1000Research*. This is a very interesting and important study, which addresses a timely and important question – in a context of a growing number of preclinical and clinical studies showing a potential efficacy of certain antidepressants, and particularly fluoxetine, in COVID-19, what dose of fluoxetine should be optimally prescribed in clinical trials to ensure a maximum of patients achieve the effective concentration resulting in 90% inhibition (EC90) of SARS-CoV-2 as reported in Calu-3 human lung cells. On a more general, and potentially more innovative front, it is one of a small (but quickly increasing) number of studies contributing to this topic and the first study to the reviewer’s knowledge addressing this specific pharmacokinetic issue, which is an important area of inquiry with major implications in this context of pandemic. There is much to like about this manuscript. The results are presented clearly, the methods are sound, the discussion follows well from it, and the manuscript is very well written. Below are several points that would strengthen the submission:

**Title:**

- A minor suggestion would be a modification of the title to increase the interest to this important study, such as “Fluoxetine pharmacokinetics and tissue distribution suggest a possible therapeutic role in COVID-19 at usual antidepressant dose”.

 

**Introduction:**

- The introduction section should be updated and include a description of results from recent studies focused on the association between antidepressant use and the course of COVID-19, which would be very helpful to readers to understand the clinical context of this study and why this study (and its results) are important. In addition to the medRxiv one, the reference to the published article of Hoertel N *et al*. should be cited (Hoertel N *et al.* (2021 ^1^)). This association/potential effect of certain antidepressants in COVID-19 has been confirmed in several other observational studies (Diez-Quevedo *et al.* (2021 ^2^), Hoertel *et al.* (2021 ^3^), Hoertel *et al. *(2021 ^4^)), two clinical trials (1 RCT and 1 open-label study) with the antidepressant fluvoxamine (which is, like fluoxetine, a functional inhibitor of sphingomyelinase acid and a S1R agonist) (Lenze *et al.* (2020 ^5^), Seftel & Boulware (2021 ^6^)), and other preclinical studies (Dechaumes *et al*. (2021 ^7^), Fred *et al.* (2021 ^8^)). Results from all those studies, increasing the interest in the present submission, should be recognized and briefly summarized in the Introduction.   
- As evoked in the discussion, potential underlying mechanisms include antiviral effect of certain antidepressants, and particularly fluoxetine, through the inhibition of the acid sphingomyelinase/ceramide system, which may play a central role in COVID-19, as suggested by preclinical studies (Carpinteiro *et al. *(2020 ^9^), and Carpinteiro *et al. *(2021 ^10^)) and observational studies (Hoertel N *et al.* (2021 ^3^), Darquennes G *et al. *(2021 ^11^), Hoertel *et al. *(2021 ^4^)), as well as anti-inflammatory properties through sigma-1-receptor agonist effect of certain antidepressants, and particularly fluoxetine and fluvoxamine (Köhler CA *et al. *(2018 ^12^), Rosen D *et al*. (2019 ^13^), Sukhatme VP *et al. *(2021 ^14^), Roumestan C *et al. *(2007 ^15^)). Moreover, a recent study (Marín-Corral *et al. *(2021 ^16^)) showed that ceramide plasma concentration is associated with inflammatory markers and clinical outcomes in COVID-19 patients. I think that results from all those studies should also be recognized and shortly summarized in the Introduction.   
- The abstract might include some of these points listed above.

** **


**Results:**

- Importantly, results from that study can help guide the choice of the right dose to use in COVID-19 clinical trials. I would strongly recommend the author to present in the result section as well as in the abstract (for main findings) for each following dose: 20 mg/d, 30mg/d (which is also interesting for clinical tolerability purpose), 40 mg/d and 60 mg/d the percentage of patients reaching a) EC90 and b) EC50 at (i) 1 day, (ii) 2 days, and (ii) 3 days. In addition, given that 40 mg/d might lead to increased risk of mild side effects among older adults and that at the same time reaching the efficient dose as fast as possible is desirable given the potential antiviral properties of fluoxetine, would a loading dose of 30 mg or 40 mg the first or two first days, followed by a dose of 20 mg/day, perform better than only taking 20 mg/d?
- Finally, is there a way to ensure that at no point of the 10 day treatment course, no patient (typically older adults) will reach a toxic plasma dose with the different scenarios, as I think 40 mg/d is the maximum recommended dose in the elderly population? Even if the reviewer recognizes that it may represent a substantial additional amount of work, these detailed results will certainly be of great help to clinicians to balance the risks associated for each dose and the potential benefits, and help them design ideal clinical trial.
- With the different scenarios, at the end of the 10-day treatment, how long would it take to have a  substantial decrease in the fluoxetine plasma concentration (and its metabolite norfluoxetine)? This information would give an idea of the duration of potential protection of the treatment against SARS-CoV-2 once stopped and of the required duration of medical follow-up.

 

**Conclusion:**

- Finally, with these different dose scenarios, what recommendation(s) of dose would do the author for a clinical trial including fluoxetine for COVID-19? Would it be desirable to do a loading dose then decrease to the standard 20 mg/d dose which is known to be very well tolerated in older adults? Or keeping 30 mg/d or 40 mg/d throughout the trial would be the best choice?

Is the work clearly and accurately presented and does it cite the current literature?

Yes

If applicable, is the statistical analysis and its interpretation appropriate?

Yes

Are all the source data underlying the results available to ensure full reproducibility?

Yes

Is the study design appropriate and is the work technically sound?

Yes

Are the conclusions drawn adequately supported by the results?

Yes

Are sufficient details of methods and analysis provided to allow replication by others?

Yes

Reviewer Expertise:

Psychiatry; antidepressants; methodology; biostatistics; epidemiology; sphingomyelinase; COVID-19.

I confirm that I have read this submission and believe that I have an appropriate level of expertise to confirm that it is of an acceptable scientific standard, however I have significant reservations, as outlined above.

## References

[1] : Association between antidepressant use and reduced risk of intubation or death in hospitalized patients with COVID-19: results from an observational study. *Mol Psychiatry* .2021; 10.1038/s41380-021-01021-4 10.1038/s41380-021-01021-4 33536545

[2] : Mental disorders, psychopharmacological treatments, and mortality in 2150 COVID-19 Spanish inpatients. *Acta Psychiatr Scand* .143(6) : 10.1111/acps.13304 526-534 10.1111/acps.13304 33792912PMC8250711

[3] : Association between FIASMAs and Reduced Risk of Intubation or Death in Individuals Hospitalized for Severe COVID-19: an observational multicenter study. *Clin Pharmacol Ther* .2021; 10.1002/cpt.2317 10.1002/cpt.2317 34050932PMC8239599

[4] : Association between Psychotropic Medications Functionally Inhibiting Acid Sphingomyelinase and reduced risk of Intubation or Death among Individuals with Mental Disorder and Severe COVID-19: an Observational Study. *medRxiv* .2021; 10.1101/2021.02.18.21251997 10.1101/2021.02.18.21251997

[5] : Fluvoxamine vs Placebo and Clinical Deterioration in Outpatients With Symptomatic COVID-19. *JAMA* .2020;324(22) : 10.1001/jama.2020.22760 10.1001/jama.2020.22760 33180097PMC7662481

[6] : Prospective Cohort of Fluvoxamine for Early Treatment of Coronavirus Disease 19. *Open Forum Infect Dis* .2021;8(2) : 10.1093/ofid/ofab050 ofab050 10.1093/ofid/ofab050 33623808PMC7888564

[7] : Fluoxetine Can Inhibit SARS-CoV-2 In Vitro. *Microorganisms* .2021;9(2) : 10.3390/microorganisms9020339 10.3390/microorganisms9020339 33572117PMC7914627

[8] : Antidepressant and antipsychotic drugs reduce viral infection by SARS-CoV-2 and fluoxetine show antiviral activity against the novel variantsin vitro. *bioRxiv* .2021; 10.1101/2021.03.22.436379 10.1101/2021.03.22.436379 PMC880940835126106

[9] : Pharmacological Inhibition of Acid Sphingomyelinase Prevents Uptake of SARS-CoV-2 by Epithelial Cells. *Cell Rep Med* .2020;1(8) : 10.1016/j.xcrm.2020.100142 100142 10.1016/j.xcrm.2020.100142 33163980PMC7598530

[10] : Inhibition of acid sphingomyelinase by ambroxol prevents SARS-CoV-2 entry into epithelial cells. *J Biol Chem* .2021; 10.1016/j.jbc.2021.100701 100701 10.1016/j.jbc.2021.100701 33895135PMC8062550

[11] : Association between Functional Inhibitors of Acid Sphingomyelinase (FIASMAs) and Reduced Risk of Death in COVID-19 Patients: A Retrospective Cohort Study. *Pharmaceuticals (Basel)* .2021;14(3) : 10.3390/ph14030226 10.3390/ph14030226 33799977PMC7998809

[12] : Peripheral Alterations in Cytokine and Chemokine Levels After Antidepressant Drug Treatment for Major Depressive Disorder: Systematic Review and Meta-Analysis. *Mol Neurobiol* .2018;55(5) : 10.1007/s12035-017-0632-1 4195-4206 10.1007/s12035-017-0632-1 28612257

[13] : Modulation of the sigma-1 receptor–IRE1 pathway is beneficial in preclinical models of inflammation and sepsis. *Science Translational Medicine* .2019;11(478) : 10.1126/scitranslmed.aau5266 10.1126/scitranslmed.aau5266 PMC693625030728287

[14] : Fluvoxamine: A Review of Its Mechanism of Action and Its Role in COVID-19. *Front Pharmacol* .2021;12: 10.3389/fphar.2021.652688 652688 10.3389/fphar.2021.652688 33959018PMC8094534

[15] : Anti-inflammatory properties of desipramine and fluoxetine. *Respir Res* .2007;8: 10.1186/1465-9921-8-35 35 10.1186/1465-9921-8-35 17477857PMC1876225

[16] : Metabolic Signatures Associated with Severity in Hospitalized COVID-19 Patients. *Int J Mol Sci* .2021;22(9) : 10.3390/ijms22094794 10.3390/ijms22094794 33946479PMC8124482
